# When cultures clash: Links between perceived cultural distance in values and attitudes towards migrants

**DOI:** 10.1111/bjso.12455

**Published:** 2021-05-09

**Authors:** Katja Albada, Nina Hansen, Sabine Otten

**Affiliations:** ^1^ Department of Social Psychology University of Groningen Groningen The Netherlands

**Keywords:** cultural distance, host‐society, intergroup relations, migrants, outgroup attitudes, values

## Abstract

Migration elicits mixed reactions from the host‐society. Negative responses towards migrants seem to emerge when migrants are perceived as culturally different. We investigated when and why perceived cultural distance (PCD) is associated with negative migrant attitudes by focussing on differences in cultural values. We expected that PCD in *social values* (focus on relationships and society) should be more strongly associated with attitudes towards migrants than personal values (individual needs and gains) and should be mediated by symbolic threat. In two quasi‐experimental studies (*N* = 200, *N* = 668) with Dutch participants (host‐society), we simultaneously tested effects of respondents’ perception of Dutch values, their perceptions of migrant values (of Moroccan, Syrian, Polish ethnic origin), and PCD between Dutch‐migrant value on attitudes. For all migrant groups, PCD in social values was associated with more negative attitudes, less tolerance, and less policy support regarding migrants; this was mediated by symbolic threat. These links were weaker for personal values.

## Background

In 2015, the number of migrants coming to Europe suddenly increased (UNHCR, 2019). Although some people are willing to welcome and support refugees, others are reluctant to accept them as residents and see them as a threat (e.g., Albada, Hansen, & Otten, 2021; SCP, 2016). A considerable number of people in Europe are concerned about migration and intolerant towards refugees and migrants (European Social Survey, 2017). This seems to especially apply when refugees and migrants are perceived to be very different compared to members of a host‐society (e.g., Mahfud, Badea, Verkuyten, & Reynolds, 2018). The more distinct members of another cultural group are perceived, the larger the perceived cultural distance (PCD; e.g., Babiker, Cox, & Miller, 1980). Greater PCD between members of a host‐society and migrants is associated with more negative attitudes towards migrants (Mahfud et al., 2018). However, we know very little about *which specific aspects of PCD* may evoke negative migrant attitudes. The current research sets out to investigate *when* and *why* members of a host‐society may perceive PCD, and how this is associated with negative attitudes towards migrants. It aims at providing a more systematic understanding of the role PCD in attitudes towards migrants by, first, differentiating in PCD in *social values* (i.e., with respect to relationships and society) versus *personal values* (i.e., individual needs and gains; Schwartz et al., 2012), second, focussing on three distinct groups of immigrant origin in the Netherlands, and third, investigating symbolic threat as mediator.

### Perceived cultural distance

In psychology, culture is defined as a multilayered, interacting, dynamic system of ideas, institutions, interactions, and individuals (e.g., Hamedani & Markus, 2019). This system becomes internalized as people socialize in certain environments; it is reflected in norms, values, and morals that become part of people’s cultural identity (Hall, 1992). Culture is at play on different levels (i.e., societal, institutional, interpersonal, and individual), which mutually and dynamically influence each other (Markus & Kitayama, 2010; Shweder, 2003). Hence, culture is more than a fixed set of beliefs residing inside a group of individuals.

However, lay people’s understanding of culture can be oversimplified and viewed as a fixed entity, which promotes stereotyping (Levy, Plaks, Hong, Chiu, & Dweck, 2001). Indeed, cultural background is one of the most essentialized social categories, which means that people may believe that culture represents a foundational, fixed core with unchanging properties that make group members what they are (Haslam, Rothschild, & Ernst, 2000; Prentice & Miller, 2007). As a result, people overestimate similarities within and differences between cultural groups (e.g., Adams & Markus, 2004).

The idea that cultural differences are an important factor in intergroup relations is not new. Since long, researchers have argued that such differences may trigger prejudice (e.g., Allport, Clark, & Pettigrew, 1954; Pettigrew, 1998), are problematic for acculturation (Berry, 1992; Piontkowski, Rohmann, & Florack, 2002), and may give rise to intergroup conflict (Riek, Mania, & Gaertner, 2006). Research suggests that PCD may result in negative consequences. For example, greater PCD was associated with more anxiety and more medical consultations among foreign students in the United Kingdom (Babiker et al., 1980), lower levels of well‐being and psychological adaptation among sojourns in the United Kingdom (Demes & Geeraert, 2014), and lower psychological and socio‐cultural adaption among exchange students in Russia (Suanet & van de Vijver, 2009). Furthermore, higher PCD related to higher identification with the country of origin among migrants in Australia (Nesdale & Mak, 2003), more desire for assimilation, separation, and marginalization, and less desire for integration among host‐society members (German, Swiss, and Slovakian) and migrants (Turkish and former Yugoslavian in Switzerland and Germany; Piontkowski, Florack, Hoelker, & Obdrzálek, 2000). In addition, more PCD was associated with more negative attitudes towards migrants among Dutch and French host‐society members (Mahfud et al., 2018). Previous research on the consequences of PCD mainly studied the minority perspective (few focussed on the majority perspective; e.g., Mahfud et al., 2018).

Perceived cultural distance has mostly been investigated as broad construct, containing a relatively large set of cultural differences (e.g., Bierwiaczonek & Waldzus, 2016); a list of possible differences (such as in language, food, religion, and values) is summarized into one general score without differentiating specific aspects. Other studies have focused on cultural groups, for which different levels of PCD were assumed, but not measured. For example, Briones, Verkuyten, Cosano, and Tabernero (2012) showed that Moroccan adolescents had weaker psychological adaptation (life satisfaction, social support, social self‐efficacy) and experienced more discrimination than Ecuadorian adolescents in Spain. These authors argued that adolescents from Moroccan descent have a greater cultural distance to the Spanish host‐society compared to Ecuadorian adolescents due to larger differences in language and religious background.

The concordance model of acculturation (Piontkowski et al., 2002) assumes that a perceived mismatch may be associated with negative outgroup attitudes. This model specifically focusses on differences in acculturation orientations. It proposes that the more migrants are perceived to have different acculturation orientations than the host‐society expects, the more threatening and negative the intergroup relation is evaluated. Similarly, the current research assumes that perceived differences in values between host‐society and migrants may lead to negative attitudes towards migrants.

It is important to note that cultures can be similar on objective measures [such as Hofstede’s (2001) cultural dimensions] but still be *perceived* as dissimilar. Indeed, objective and perceived measures of cultural distance not always correlate with each other (Bierwiazonek & Waldzus, 2016; Suanet & van de Vijver, 2009). We therefore focus on PCD; we assume that it is host‐society members’ *perceptions* of migrants that will likely shape the intergroup relation.

To conclude, previous research on intergroup relations (e.g., Mahfud et al., 2018) and acculturation (e.g., Piontkowski et al., 2002) indicated that greater PCD was associated with more negative intergroup relations. However, this research mostly studied PCD as a broad construct either focusing on a general score or comparing groups considered to differ in cultural distance. However, as we will argue below, some cultural differences may be more relevant in intergroup relations than others. *Which aspects of PCD* may be more strongly associated with negative attitudes and *why* remains unclear (see Bierwiaczonek & Waldzus, 2016; Briones et al., 2012, for similar conclusions).

### Perceived cultural distance and intergroup relations

People can dislike certain cultural practices of outgroups, yet tolerate those groups without these differences leading to problematic intergroup relations (Verkuyten, Yogeeswaran, & Adelman, 2019). Indeed, perceiving specific cultural differences need not result in negative outgroup attitudes per se (Gibson, 2006). For example, people can disapprove of specific cultural symbols (e.g., headscarves) without holding negative attitudes towards the group (Muslims) associated with these cultural symbols (Van der Noll, 2010). Moreover, when people reject a certain cultural practice, they not necessarily reject *all* practices of this group (Van der Noll, 2014; Van Doorn, 2015).

People are motivated to see outgroups as distinct from their ingroup (Brewer, 1993; Jetten, Spears, & Postmes, 2004; Tajfel & Turner, 1979). Differentiating the ingroup from outgroups can create a sense of group identity, but not necessarily intergroup conflicts (e.g., Mummendey & Otten, 2011). Accordingly, there is good reason to assume that PCD does not always lead to negative outgroup attitudes. There are instances where people actively seek out and enjoy cultural differences. For example, many people enjoy consuming social media from cultures that are distinct (‘K‐pop’ Beak, 2015), enjoy foreign dramas (Beak & Kim, 2016), or prefer travelling to exotic countries to venture away from daily routine and explore other cultures (Bi & Lehto, 2018).

### Perceived cultural distance in values

Cultures (can) differ in many ways; an important aspect herein is cultural values. Values are lasting beliefs about what is important and desirable (Schwartz et al., 2012). They offer guidance in how to behave and can provide an understanding of the cultural environment (Triandis, 1994). They can systematically map a large spectrum of cultural differences; there is extensive research showing that values have a universal circular structure across cultures (Schwartz et al., 2001). According to the circumplex model (Schwartz, 1992), values can be differentiated into social and personal values, which can be organized along two orthogonal dimensions: *self‐transcendence* (social) versus *self‐enhancement* (personal) and *conservation* (social) versus *openness* (personal). Social values focus on concerns for the welfare of others, on how to behave in society, how to treat others, and the extent to which people should follow the societal rules. Social values encompass benevolence and universalism under the higher‐order value self‐transcendence; and conformity, security, and tradition under the higher‐order value conservation. Personal values are about promotion of the self and personal interests or needs. They encompass power and achievement under the higher‐order value self‐enhancement; and self‐direction, hedonism, and stimulation under the higher‐order value openness.

A recent study offered first systematic insights in the impact of perceived value differences on prejudice towards migrants (Wolf, Weinstein, & Maio, 2019). British students indicated their own value endorsement and the perceived value endorsement of Muslim migrants, economic migrants, and refugees based on the two dimensions of self‐transcendence/self‐enhancement and openness/conservation (Schwartz, 1992). The researchers investigated the link between the perceived dissimilarity of values (self‐immigrant) on prejudice. They found that British students holding higher conservation values were more negative towards migrants when they perceived migrants to value openness more. The current research took a different perspective and differentiated between social and personal values. We focussed on perceived dissimilarities in value endorsement of the ingroup (the host‐society) versus the outgroup (migrant group) to gather more insights about the intergroup relationship.

Social values play an important role in the functioning of societies (Campbell, 1975; Parsons, 1951). Social rules on how to behave can help avoid conflicts between individuals and groups and may promote harmonious relations (Boyd & Richerson, 2002; Horne, 2001; Sherif, 1936). Moreover, caring about others and their welfare assures that most members of society can interact harmoniously with people that are both closely (e.g., family members) and loosely related (e.g., citizens in a country). Hence, if host‐society members perceive a distance between the endorsement of social values by the host‐society and a migrant group, this may substantially affect evaluations of migrants. In the current research, we simultaneously tested effects of respondents’ perception of Dutch values, their perceptions of migrant values (Moroccan, Syrian, Polish), and PCD between these two on attitudes. We hypothesize that for members of the host‐society, greater PCD in social values is associated with more negative attitudes towards migrants (*Hypothesis 1*). We expected this to be a general process applying to different cultural outgroups and attitudes.

In the private sphere, differences in personal values can be maintained without necessarily influencing others (Forst, 2013). Personal needs underlie personal values (Schwartz & Bardi, 2001). These needs can vary across individuals and therefore may play a smaller role in intergroup relationships than social values, which are presumably based on universal needs. Moreover, because social values guide how people interact with each other, they may be more likely to surface in social interactions (Schwartz et al., 2012), making these differences more difficult to ignore than other cultural differences (e.g., different food preferences). PCD in personal values could also be linked to attitudes towards migrant, but we argue that social values might show a stronger link in the current intergroup context. Hence, we expected that PCD in social values should be more strongly associated with negative attitudes towards migrants than PCD in personal values (*Hypothesis 2*).

Previous research indicated that symbolic threat—the belief that outgroups challenge the ingroup´s values and worldviews—is a strong predictor of negative outgroup attitudes (Riek et al., 2006; Stephan & Stephan, 2000). However, we still know little about *which* differences may be experienced as threating. Symbolic threat differs from PCD as it has a clear negative valence, whereas PCD is solely about the amount of differences people may observe without evaluating its valence; hence, in principle a large PCD could be perceived as fascinating as well as threatening. Accordingly, the impact of PCD on outgroup attitudes should depend on the extent to which it elicits symbolic threat. We, thus, expected that symbolic threat should mediate the relationship between PCD in social values and attitudes towards migrants (*Hypothesis 3*).

## The current research

The present research addresses the question of *when* and *why* PCD could explain negative attitudes towards migrants. We extend previous research in three ways: First, we investigated PCD by focussing on and differentiating between *social* and *personal* values to gain new insights, *which* perceived differences might be more strongly related to negative outgroup attitudes.

Second, we zoomed in on how members of the Dutch host‐society perceived differences between the Dutch culture and three different groups of immigrant origin, namely Moroccan, Syrian, and Polish migrants in the Netherlands. *People of Moroccan origin* are the largest non‐Western migrant group^1^ having settled in the Netherlands since the sixties and consisting of first, second, and third generations, who may identify as Dutch, Moroccan, or both. *People of Syrian origin* were forced to flee their country due to the civil war; they are currently the largest ‘new’ migrant group in the Netherlands. *People of Polish origin* are one of the largest Western migrant groups. Their number has substantially increased since EU borders were opened in 2004. Many of them migrate temporarily to the Netherlands to find work (SCP, 2018). Previous research in this context has mostly focused on broader categories such as refugees (e.g., Greenhalgh & Watt, 2015), Muslim migrants, economic migrants, and refugees (e.g., Wolf et al., 2019). However, evaluations of specific migrant groups can differ substantially (e.g., Lee & Fiske, 2006). We selected the abovementioned three groups as they represent three large and quite different groups of immigrant origin in the Netherlands. We employed a quasi‐experimental (between‐subjects) research design to test the generalizability of our hypotheses with respect to host‐society members’ perceptions of three different groups of immigrant origin. More precisely, we simultaneously tested the effects of respondents’ perception of Dutch values (Dutch), their perceptions of migrant values (migrant; Moroccan, Syrian, Polish), and PCD (Dutch‐migrant) in values on attitudes.

Third, to increase the validity of our research, we first tested our hypotheses with both a student sample (Study 1) and a more representative sample of the Dutch host‐society (Study 2). Furthermore, we included different measures to assess outgroup attitudes and test the mediating influence of symbolic threat (Study 2). Finally, summarizing our research findings, we conducted an internal mini‐meta‐analysis on the relationship between PCD in values and migrant attitudes across the two studies.

## STUDY 1

## Method

### Participants, design, and procedure

The required sample size (*n* = 55 per condition) was determined using G*power (multiple linear regression, medium effect size, .80 power, .05 alpha‐level; Faul, Erdfelder, Lang, & Buchner, 2007). In total, 200 Dutch students from the university’s online participant pool (75% female, mean age = 21.66, *SD* = 3.94) participated. The majority was non‐religious (76%). Participants were politically rather left‐wing oriented [*M* = 2.09, *SD* = 2.16, on a 1 (l*eft‐wing*) to 7 (*right‐wing*) scale]. No participants were excluded. After giving informed consent, using a quasi‐experimental (between‐subject) design, participants were randomly assigned to one of three conditions: Moroccan migrants (*n* = 70), Syrian migrants (*n* = 63), or Polish migrants (*n* = 67). All participants completed the same online questionnaire, which differed with respect to the target migrant group per condition. Participants first answered demographic questions (e.g., age, gender) and next questions about their perception of Dutch values, their perceptions of values of the respective migrant group, attitude measures, and finally additional demographic information (e.g., political orientation). Afterwards, participants were thanked, debriefed, and received four Euros for compensation.

### Measures

Cronbach’s alphas per migrant condition can be found in the Appendix S1. Scales ranged from 1 (*completely disagree*) to 7 (*completely agree*), unless otherwise indicated.

#### Perceived cultural distance in values

We used the refined Portrait Values Questionnaire (PVQ5X; Schwartz et al., 2012) to measure PCD in values. We adapted this scale in two ways. First, we excluded the value universalism‐nature, which was not relevant in the current research; it assesses people’s biospheric values, which are not clearly personal nor social values. Moreover, we excluded security‐societal and security‐personal values, because these items did not fit to the current context. Second, we measured each value with two instead of three items to minimize fatigue in participants. We selected the items with the highest factor loadings according to Schwartz et al., (2012). Participants first answered questions about their perception of Dutch values (e.g., being very successful is important to Dutch people) and next their perceptions of values of one migrant group (Moroccan, Syrian, Polish).

In total, participants answered 32 items about their perception of the Dutch culture and 32 items about their perception of one migrant group; two items for each of the 16 values which can be clustered in four subscales. The subscales self‐transcendence (benevolence – dependability, benevolence – caring, universalism – concern, and universalism – tolerance) and conservation (tradition, conformity – rules, conformity – interpersonal, and humility) are social values (Schwartz et al., 2012). The subscales openness (self‐direction – thought, self‐direction – action, stimulation, and hedonism) and self‐enhancement (achievement, power resources, power dominance, and face) are personal values. The reliabilities of these subscales were moderate to good (range α’s = .66–.84).

#### General perceived cultural distance (GPCD)

Next, using a slide‐bar, participants indicated the general distance they perceived between the Dutch and the migrant culture. The slide bar ranged from 0 (*no difference*) to 100 (*very large difference*). We included GPCD for descriptive purposes to compare the three migrant groups in general PCD evaluations and to check whether the specific measures of PCD in values were more strongly linked to migrant attitudes than such general PCD measure.

#### Attitudes towards migrants

We measured attitudes towards migrants on three dimensions: sociability, competence, and morality (e.g., Leach, Ellemers, & Barreto, 2007). Participants rated to what extent nine characteristics applied to the migrant group (e.g., likeable, competent, and trustworthy). Results of each subscale were comparable; hence, in the following, we report the results of the overall scale (α = .87).

## Results

First, we tested for differences in the evaluations of the migrant groups with one‐way ANOVAs (see Table 1). Syrian migrants were evaluated more positively than Polish migrants. Moreover, general PCD was higher for Moroccan and Syrian migrants than for Polish migrants.

**Table 1:** Study 1: Evaluations of the migrant groups

	Migrant groups			ANOVA statistics	
	Moroccan *M* (*SD*)	Syrian *M* (*SD*)	Polish *M* (*SD*)	*F* (*η* ^2^)	Condition differences*
GPCD^a^	64.86 (14.15)	65.05 (16.76)	48.15 (19.65)	21.84 (0.18)**	AB > C
Migrant attitudes^b^	4.99 (0.85)	5.23 (0.71)	4.84 (0.57)	4.66 (0.05)*	B > C

Note

GPCD is an abbreviation for general perceived cultural distance.

^a^ Measured on a 100‐point scale

^b^ Measured on a 7‐point scale. Higher migrant attitudes indicate more positive attitudes. One‐way ANOVAs with *df* (2, 198). Bonferroni corrections were used

* *p* < .05.

** *p* < .01.

### Polynomial regression

**Figure 1:**
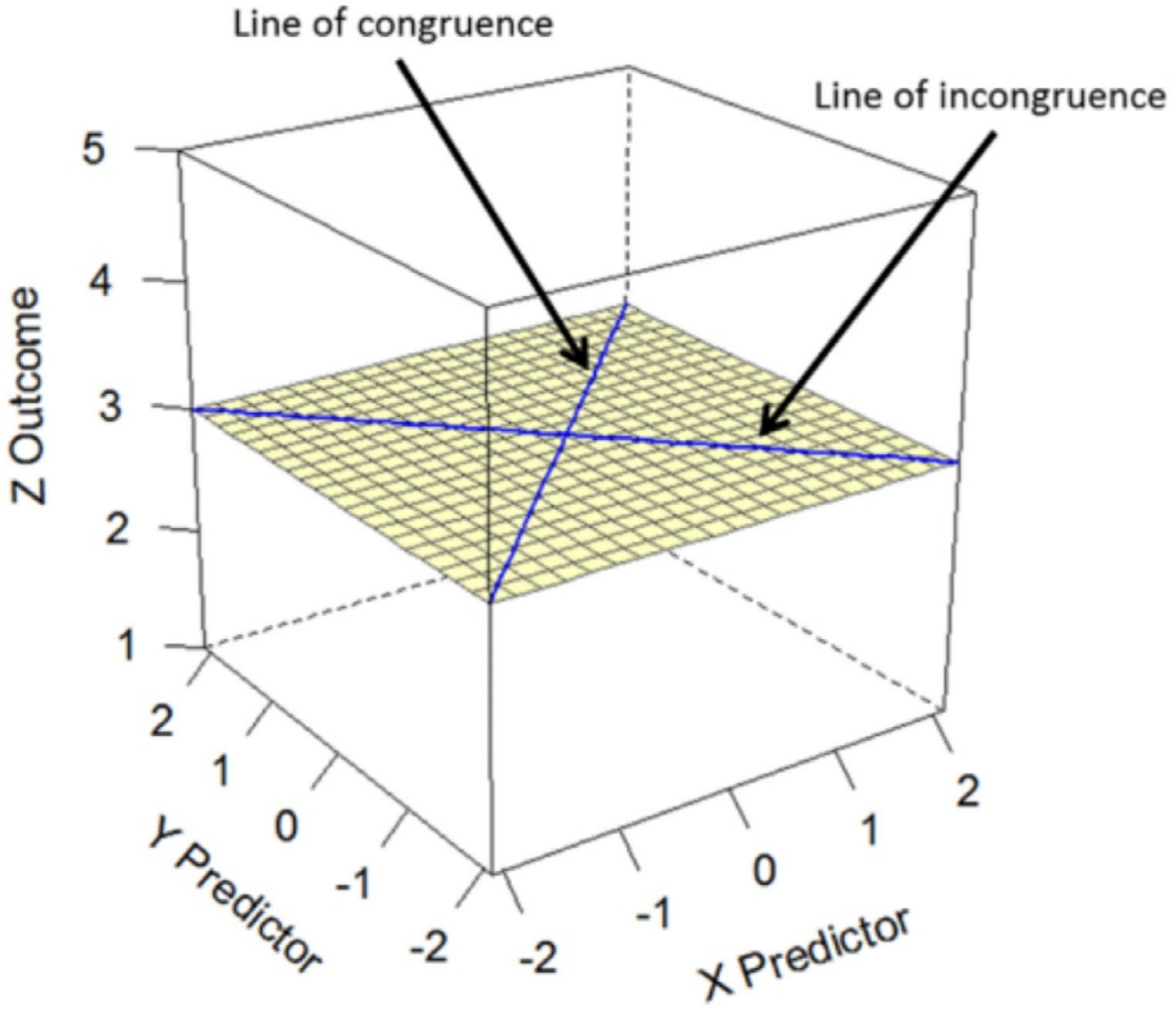
Response surface with labels (Barranti et al., 2017). *Note*. When the lines are flat as in the current figure, effects are non‐significant.

We conducted polynomial regression analyses, which has two main advantages over regular regression analyses. First, polynomial regression can test to what extent the relation between PCD in values and migrant attitudes is due to respondents’ perception of Dutch values, their perceptions of migrant values (simple and quadric slopes), and their combined (interaction) effect (Barranti, Carlson, & Côté, 2017; Wolf et al., 2019). Second, this analysis tests for dissimilarity effects, that is, whether more perceived distance between the perception of Dutch values and the perception of migrant values is associated with more negative migrant attitudes (Edwards, 2002). It can identify whether the PCD in values plays a unique role in predicting attitudes towards migrants. The regression coefficients are plotted in a three‐dimensional space, and the surface is analysed (Response Surface Analyses, RSA) to investigate *where specifically* dissimilarity effects occur (see Figure 1). The interpretations of dissimilarity effects focus on the RSA rather than on the regression coefficients and show effects not detectible by regular regressions (e.g., Barranti et al., 2017). We conducted the analyses in SPSS and RSA in Excel (Shanock, Baran, Gentry, Pattison, & Heggestad, 2010). All independent variables were centred around the scale midpoint. We conducted separate analyses for each value subscale (self‐transcendence, conservation, openness, self‐enhancement).

#### PCD in social values

**Figure 2:**
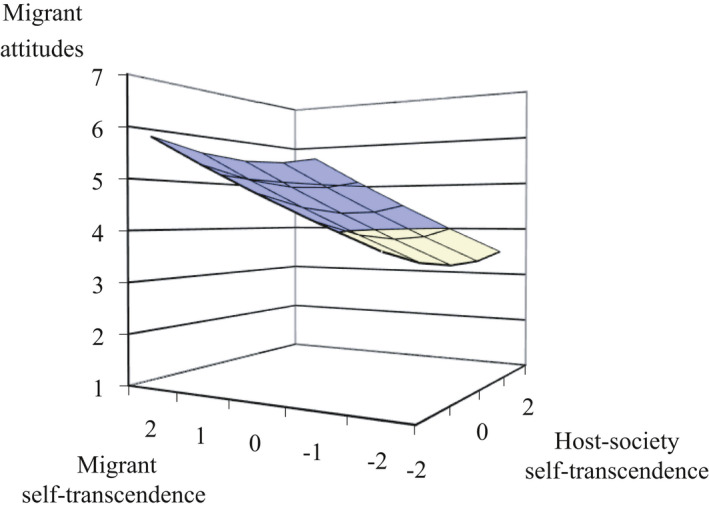
Response surface of PCD in self‐transcendence in the Moroccan migrant condition of Study 1. *Note*. PCD is an abbreviation of perceived cultural distance. Colour in the graph is only intended to improve the graph’s three‐dimensional perspective. The lines of congruence (*a*_1_) and incongruence (*a*_3_) are steep and linear (also see Figure 1), and the overall graph tends to be straight rather than curved indicating that the relationship between PCD and migrant attitudes is linear (there is a slight curve based on the *a*_2_ and *a*_4_ coefficients, suggesting non‐linearity, but these were non‐significant which is not taken into account when plotting the graph).

We tested whether greater PCD in social values was associated with more negative migrant attitudes (*Hypothesis 1*). We report results for the overall sample and then per migrant condition (see Tables 2 and 3). First, we examined the explained variance to inspect how strongly PCD in social values was associated with migrant attitudes; the two social values (self‐transcendence and conservation) explained a considerable proportion of variance (27% and 23%). Second, for both social values, the simple slopes for perceived migrant’s values were significant (*b*_2_ = 0.43 and *b*_2_ = 0.51, *p*’s < .01); the less people perceived migrants to endorse social values the more negative their attitudes towards migrants. No other slope was significant (all *b*’s < 0.31). This indicates that the relationship between PCD in social values and migrant attitudes can mostly be explained by the perceived value endorsement of migrants and not by the perceived endorsement of the host‐society. Third, most importantly, the line of incongruence (*a*_*3*_) in the RSA was significant (both *p*’s < .05) for both social values, indicating a dissimilarity effect (in other words, PCD). The more migrants were perceived to endorse social values *less* strongly than the host‐society, the more negative peoples’ attitudes. Importantly, due to only the migrant slopes, *a*_*1*_ and *a* *_3_* being significant we can conclude that the relationship between PCD in social values and migrant attitudes is linear (see Figure 2 and Appendix S1), meaning that the direction of the perceived differences mattered and not necessarily absolute differences.

**Table 2:** Study 1: Descriptive statistics and Pearson’s correlations between variables

	*M* (SD)	GPCD	Migrant attitudes
Moroccan migrants			
GPCD	64.86 (14.15)	−	−.27*
PCD self‐transcendence	−0.22 (0.96)	.26*	−.33**
PCD conservation	−0.99 (1.32)	.13	−.37**
PCD openness	1.59 (1.01)	.19	−.06
PCD self‐enhancement	−0.17 (1.19)	−.19	.05
Syrian migrants			
GPCD	65.05 (16.76)	−	−.26*
PCD self‐transcendence	−0.63 (0.96)	.22	−.40**
PCD conservation	−1.45 (1.02)	.18	−.34**
PCD openness	1.41 (0.99)	.21	−.06
PCD self‐enhancement	0.66 (1.14)	−.16	.20
Polish migrants			
GPCD	48.15 (19.65)	−	.−31*
PCD self‐transcendence	−0.03 (0.93)	.15	−.24†
PCD conservation	−0.90 (1.17)	.33**	−.11
PCD openness	1.33 (1.00)	−.03	−.16
PCD self‐enhancement	0.24 (1.22)	−.11	.02

Note

GPCD is an abbreviation for general perceived cultural distance and PCD for perceived cultural distance, which are difference scores (host‐society values minus migrant values). Lower migrant attitudes indicate more negative attitudes (e.g., perceiving migrants to endorse self‐transcendence less than the host‐society is associated with more negative migrant attitudes).

* *p* < .05

** *p* < .01

† *p* = .05.

**Table 3:** Study 1: Polynomial regression of migrant attitudes by PCD in values

	Slopes *b* (*SE*)						Response Surface Test			
	*b*_1_	*b*_2_	*b*_3_	*b*_4_	*b*_5_	$R_{\text{adj}}^{2}$	*a*_1_	*a*_2_	*a*_3_	*a*_4_
Overall sample	NL	M	NL^2^	NLM	M^2^					
Self‐transcendence	0.01 (.17)	0.43 (.16)**	0.01 (0.08)	0.01 (0.09)	0.05 (.06)	.27**	0.44	0.07	−0.42*	0.04
Conservation	0.04 (.11)	0.51 (.12)**	−0.08 (0.07)	0.03 (.07)	−0.03 (0.06)	.23**	0.55**	−0.09	−0.47**	−0.14
Openness	−0.14 (.36)	0.31 (.28)	0.10 (.11)	−0.08 (.14)	0.06 (0.05)	.03*	0.17	0.07	−0.45	0.24
Self‐enhancement	−0.02 (9.14)	−0.10 (.12)	0.06 (.06)	0.10 (0.07)	0.03 (.05)	.00	−0.12	0.10	0.09	0.08
Moroccan migrants										
Self‐transcendence	−0.03 (.34)	0.54 (.34)	0.01 (.15)	0.07 (.19)	0.02 (.12)	.22**	0.51	0.10	−0.57	0.07
Conservation	−0.05 (.18)	0.48 (.20)*	−0.11 (.11)	0.10 (.12)	0.04 (.10)	.26**	0.43	0.02	−0.53*	−0.17
Openness	−2.08 (1.18)	0.41 (.51)	0.65 (.34)	−0.15 (.25)	0.08 (.11)	.02	−1.67	0.58	−2.48	0.88*
Self‐enhancement	−0.81 (.37)*	−0.69 (.40)	0.19 (.13)	0.44 (.21)*	0.12 (.14)	.02	−1.51*	0.75*	−0.12	−0.14
Syrian migrants										
Self‐transcendence	−0.05 (.34)	0.45 (.36)	0.06 (.17)	−0.06 (.18)	0.03 (.12)	.19**	0.40	0.03	−0.50	−0.09
Conservation	−0.28 (.28)	0.44 (.11)*	−0.16 (.16)	0.14 (.18)	−0.19 (.13)	.14*	0.54	−0.22	−1.10*	−0.49
Openness	−0.72 (.73)	0.06 (0.48)	0.27 (.21)	0.02 (.25)	−0.04 (.07)	.00	−0.66	0.25	−0.79	−0.21
Self‐enhancement	0.08 (.24)	0.01 (.25)	0.05 (.10)	−0.27 (.17)	0.19 (.11)	.05	0.09	−0.03	−0.08	0.52*
Polish migrants										
Self‐transcendence	0.05 (.24)	0.12 (.23)	0.02 (.13)	0.05 (.13)	0.16 (.10)	.26**	0.17	0.22	−0.07	0.19
Conservation	0.25 (.14)	0.34 (.19)^1^	0.06 (.10)	−0.01 (.13)	−0.02 (.10)	.15*	0.61*	0.03	−0.11	0.05
Openness	0.39 (.35)	0.32 (.41)	−0.11 (.12)	−0.09 (.20)	0.23 (.08)**	.18*	0.70	0.03	0.07	0.21
Self‐enhancement	−0.10 (.16)	0.02 (.12)	0.08 (.09)	0.02 (.09)	0.07 (.06)	.05	0.01	0.17	−0.03	0.13

Note

NL is an abbreviation for the Netherlands and indicates the perceptions of Dutch host‐society’s values. M is an abbreviation for Migrant and indicates the perceptions of migrant values. The significance of the slopes and response surface coefficients is determined by error estimates; hence, some larger coefficients may not be significant.

* *p* < .05

** *p* < .01. See Appendix S1 for the exact *p*‐values.

Closer inspection of the three migrant conditions provides similar results. First, in all conditions, the two PCD in social values explained a considerable proportion of the variance in migrant attitudes (between 15% and 26%, *p*’s < .05). Second, in the Moroccan and Syrian migrant condition, the slope for perceived migrants’ endorsement of *conservation* values was significant (*b*_2_ = 0.48 and *b*_2_ = 0.44, *p*’s < .05). Third, in the Moroccan and Syrian conditions, the line of incongruence (*a*_3_) of conservation was significant (*a*_3_ = −0.53 and *a*_3_ = −1.10, *p*’s < .05), indicating a dissimilarity effect. In the Polish condition, we found similar results; however, the slope was only marginally significant (*b*_2_ = 0.34, *p* =.059), *a*_3_ was not significant. In addition, in all conditions the coefficients regarding *self‐transcendence* showed a similar trend as *conservation*; however, they were not significant (*b*_2_ = 0.54, *b*_2_ = 0.44, and *b*_2_ = 0.12; *a*_3_ = −0.57, *a*_3_ = −0.50, and *a*_3_ = ‐0.07; explained variance was significant).

Overall, these results suggest that when people perceived migrants to endorse social values less than the host‐society, they were more negative towards migrants. This relationship was linear, meaning that people had negative attitudes towards migrants when migrants were perceived to endorse social values *less* than the host‐society across perceptions of the host‐society’s value endorsement. When migrants were perceived to endorse social values *more*, people had less negative migrant attitudes.

#### Comparison of PCD in social and personal values

Next, we tested whether PCD in *social* values was more strongly associated with migrant attitudes than PCD in *personal* values (*Hypothesis 2*; openness and self‐enhancement). First, PCD in personal values explained much less variance in attitudes towards migrants (between 0% and 3%) than PCD in social values (between 14% and 27%). Second, for personal values, in the overall sample none of the slopes nor RSA coefficients were significant. There were some significant results for the personal values in the Moroccan and Syrian migrant condition (see Table 3). For example, in the Moroccan migrant condition, the simple slope of host‐society’s endorsement of self‐enhancement, the interaction, *a*_1_ and *a*_2_ were significant. This indicated that when people perceived the host‐society to strongly endorse self‐enhancement, but perceived Moroccan migrants to do so to a lesser extent, their attitudes towards Moroccan migrants were more negative. In sum, the results for personal values were weak, inconsistent across conditions and values, and depended on the perceived host‐society’s value endorsement.

## Discussion

Altogether, Study 1 provided support for *Hypothesis 1*: when host‐society members perceived migrants to endorse social values less than the host‐society, their attitudes towards migrants were more negative. Importantly, these results did not depend on how strongly the host‐society was perceived to endorse these values. In the Polish condition, we found mixed results for the social value of self‐transcendence, and the regression coefficient for conservation was only marginally significant. It is possible that the relationship between PCD in social values and attitudes towards Polish migrants was weaker than for Moroccan and Syrian migrants and that the current sample size was too small to detect such smaller effects. Moreover, confirming *hypothesis 2*, PCD in social values was more strongly associated with attitudes towards migrants than PCD in personal values. While these results are certainly encouraging, they also ask for more research. To be able to generalize our findings, Study 2 set out to provide a replication with a more representative sample of the Dutch host‐society.

## STUDY 2

Study 2 aimed at extending Study 1 in three important ways. First, we tested our findings in a larger, more representative sample of the Dutch host‐society. Second, to test the generalizability of our findings, we added two variables to assess attitudes towards migrants: migrant policy support and intergroup tolerance. Policy support is an attitude that can have practical implications; it requires willingness of the host‐society to improve migrants’ position in society. Tolerance is an attitude that can be considered a minimal requirement for groups to coexist. Rather than striving for groups’ liking each other, tolerating each other may be a more feasible outcome (e.g., Verkuyten et al., 2019). Third, we tested whether symbolic threat would mediate the relationship between PCD in social values and migrant attitudes (*Hypothesis 3*).

## Method

### Participants, design, and procedure

We used a stricter determination for the required sample size. G*Power recommends 92 participants per condition for a multiple linear regression with five predictor variables (the slopes of a polynomial regression) for a medium effect size, power .80, and .05 alpha level (Faul et al., 2007). We decided to oversample because we tested each value separately and aimed to gain more systematic insights in the perception of Polish migrants. Via an online panel (Panel Inzicht), we recruited 727 Dutch participants. Recruitment through paid online panels can result in insufficient effort response (IER) in which participants quickly go through the survey without paying attention to the items (Curran, 2016; this was not an issue in Study 1). We excluded the fastest 5% of the participants (Maniaci & Rogge, 2014). This is a conservative exclusion method that has been shown to remove a large amount of IER without excluding too many reliable responses (Huang, Curran, Keeney, Poposki, & DeShon, 2012). Furthermore, we included a language control question; two participants, who indicated that they did not understand Dutch well, were excluded.

The final sample consisted of 689 participants, including 43 participants (6%) with incomplete responses. We did not exclude these participants, as the proportion of incomplete responses was below 10%, and they did not seem to differ from participants that fully completed the questionnaire. The sample was representative of the Dutch adult population in terms of age (*M* = 49.21, *SD* = 17.80), gender (48% male and 52% female), and education levels (31% low, 37% medium, and 32% highly educated; see CBS, 2018a, 2018b). Half of the participants (47%) indicated not to be religious [comparable with 49% reported by the Central Bureau for Statistics (2017)]. The mean of political orientation was around the midpoint of the scale (*M* = 4.15, *SD* = 1.49). Both sides of the political spectrum were well represented in the sample: 34% of the participants were right‐wing orientated, 38% were left‐wing orientated, and 24% were in between. After giving informed consent, participants were again randomly assigned to one of three conditions (Moroccan migrants *n* = 235; Syrian migrants *n* = 232; Polish migrants *n* = 222), using a between‐subject quasi‐experimental design. Participants filled in the same order of questions as in Study 1. We measured symbolic threat after the value measures and the additional attitude measures at the end (before final demographics). Afterwards, participants were thanked, debriefed, and received about one euro for compensation.

### Measures

We used the same measures as in Study 1, namely PCD in values (range: α’s = .81–.89), GPCD, migrant attitudes, symbolic threat, and added questions policy support and tolerance towards migrants. Scales ranged from 1 (*completely disagree*) to 7 (*completely agree*) unless otherwise indicated.

### Symbolic threat

Symbolic threat was measured with three items (α = .85, based on Velasco González, Verkuyten, Weesie, & Poppe, 2008).

#### Attitudes towards migrants

The *general attitudes towards migrants* scale was highly reliable (α = .96). *Policy support* was measured with three items (α = .86) that matched the current migration policy in the Netherlands (Postmes, Gordijn, Kuppens, Gootjes, & Albada, 2017). *Tolerance* was measured with four items (α = .86). Two items tapped into acceptance of the assigned migrant group (based on Schmid, Hewstone, Tausch, Cairns, & Hughes, 2009); two items referred to equal rights (based on Van der Noll, Poppe, & Verkuyten, 2010).

## Results

First, we tested for differences in the evaluations of the migrant groups with one‐way ANOVAs (see Table 4). Moroccan migrants were evaluated more negatively than Polish and Syrian migrants. Similar to Study 1, Dutch participants perceived a larger cultural distance (GPCD) for Moroccan and Syrian migrants than for Polish migrants.

**Table 4:** Study 2: Evaluations of the migrant groups by Dutch host‐society members

	Migrant groups			ANOVA statistics	
	Moroccan *M* (*SD*)	Syrian *M* (*SD*)	Polish *M* (*SD*)	*F* (*η* ^2^)	Group differences*
GPCD^a^	74.79 (17.47)	74.31 (17.84)	59.13 (20.03)	50.44 (.13)**	AB > C
Symbolic threat^b^	5.07 (1.43)	4.68 (1.50)	4.21 (1.39)	19.60 (.06)**	A > B > C
Migrant attitudes^b^	4.15 (1.19)	4.40 (1.22)	4.37 (0.98)	3.10 (.01)*	A < B^§^
Policy support^b^	3.36 (1.29)	3.68 (1.39)	3.41 (1.19)	3.77 (.01)*	A < B
Tolerance^b^	4.34 (1.38)	4.31 (1.43)	4.41 (1.28)	Not sig.	

Note

GPCD is an abbreviation for general perceived cultural distance.

^a^ Measured on a 100‐point scale

^b^ Measured on a 7‐point scale. Higher migrant attitudes indicate more positive attitudes. One‐way ANOVAs with *F*(2, 644) degrees of freedom. Bonferroni corrections were used.

* *p* < .05

** *p* < .01

§ *p* = .067.

### Polynomial regression

We conducted the same analyses as in Study 1. Because the sample size is larger, we discuss the results for each migrant condition (see Tables 5, 6, 7, 8).

**Table 5:** Study 2: Descriptive statistics and Pearson’s correlations between variables

		Correlations			
	*M* (*SD*)	GPCD	Migrant attitudes	Policy support	Tolerance
Moroccan migrants					
GPCD	74.79 (17.47)	−	−.36**	−.33**	−.38**
PCD self‐transcendence	0.70 (1.34)	.22**	−.47**	−.35**	−.33**
PCD conservation	0.39 (1.35)	.21**	−.52**	−.40**	−.35**
PCD openness	0.98 (1.07)	.18**	−.26**	−.21**	−.26**
PCD self‐enhancement	−0.23 (1.08)	−.10	−.08	.02	−.12
Syrian migrants					
GPCD	74.31 (17.84)	−	−.30**	−.34**	−.35**
PCD self‐transcendence	0.34 (1.41)	.21**	−.62**	−.57**	−.59**
PCD conservation	0.08 (1.44)	.19**	−.63**	−.53**	−.58**
PCD openness	1.02 (1.06)	.23**	−.36**	−.30	−.27**
PCD self‐enhancement	0.34 (1.09)	−.01	.06	.13	.13
Polish migrants					
GPCD	59.13 (20.03)	−	−.21**	−.21**	−.13
PCD self‐transcendence	0.65 (1.05)	.15*	−.30**	−.34**	−.32**
PCD conservation	0.52 (1.14)	.18**	−.40**	−.35**	−.44**
PCD openness	0.76 (0.89)	.10	−.21**	−.24**	−.23*
PCD self‐enhancement	0.14 (0.99)	−.04	.10	.05	−.18**

Note

GPCD is an abbreviation for general perceived cultural distance and PCD for perceived cultural distance which are difference scores (host‐society values minus migrant values). Lower migrant attitudes indicate more negative attitudes.

* *p* < .05

** *p* < .01.

**Table 6:** Study 2: Polynomial regression of migrant attitudes by PCD in values in the Moroccan migrant condition

	Slopes *b* (*SE*)					$R_{\text{adj}}^{2}$	Response Surface Test			
	b_1_	b_2_	b_3_	b_4_	b_5_		a_1_	a_2_	a_3_	a_4_
Attitudes	NL	M	NL^2^	NLM	M^2^					
Self‐transcendence	0.05 (.11)	0.74 (.08)**	−0.02 (.06)	−0.06 (.06)	0.00 (.04)	.38**	0.79**	−0.07	−0.69**	0.04
Conservation	−0.13 (.08)	0.74 (.08)**	−0.04 (.05)	−0.03 (.07)	−0.09 (.04)*	.36**	0.61**	−0.16	−0.87**	−0.10
Openness	−0.09 (.29)	0.67 (.24)**	0.06 (.11)	−0.12 (.13)	−0.04 (.07)	.11**	0.58	−0.09	−0.76*	0.14
Self‐enhancement	−0.17 (.21)	0.48 (.17)**	0.05 (.11)	0.15 (.10)	−0.26 (.07)**	.07**	0.31	−0.06	−0.64*	−0.36*
Policy support										
Self‐transcendence	0.11 (0.14)	0.55 (.10)**	−0.10 (.06)	0.02 (.07)	0.00 (.05)	.21**	0.67**	−0.08	−0.44**	0.12
Conservation	−0.16 (.10)	0.46 (.09)**	−0.09 (.07)	0.12 (.08)	−0.08 (.05)	.19**	0.30*	−0.05	−0.63**	−0.29*
Openness	0.12 (.32)	0.04 (.27)	−0.14 (.13)	0.20 (.15)	−0.05 (.08)	.05*	0.16	0.01	0.08	−0.39
Self‐enhancement	0.13 (.23)	0.15 (.18)	−0.18 (.12)	0.27 (.11)*	−0.18 (.08)*	.05**	0.28	−0.09	−0.02	−0.62**
Tolerance										
Self‐transcendence	0.15 (.15)	0.70 (.11)**	−0.02 (.07)	−0.09 (.08)	0.01 (.05)	.23**	0.86**	−0.10	−0.55**	0.08
Conservation	−0.19 (.11)	0.54 (.10)**	0.03 (.07)	−0.02 (.09)	−0.02 (.06)	.14**	0.36*	−0.02	−0.73**	0.03
Openness	0.41 (0.33)	0.66 (.28)*	−0.15 (.13)	−0.02 (.15)	−0.12 (.08)	.12**	1.07*	−0.28	−0.25	−0.25
Self‐enhancement	−0.24 (.25)	0.62 (.19)**	0.10 (.13)	0.10 (.11)	−0.27 (.09)**	.06**	0.38	−0.06	−0.86**	0.26

Note

NL is an abbreviation for the Netherlands and indicates the perception of Dutch host‐society’s values. M is an abbreviation for migrant and indicates the perception of migrant values. The significance of the slopes and response surface coefficients is determined by error estimates; hence, some larger coefficients may not be significant.

* *p* < .05

** *p* < .01. See Appendix S1 for the exact *p*‐values.

**Table 7:** Study 2: Polynomial regression of migrant attitudes by PCD in values in the Syrian migrant condition

	Slopes *b* *(SE)*					$R_{\text{adj}}^{2}$	Response Surface Test			
	b_1_	b_2_	b_3_	b_4_	b_5_		a_1_	a_2_	a_3_	a_4_
Attitudes	NL	M	NL^2^	NL*M	M^2^					
PCD self‐transcendence	−0.18 (.07)*	0.89 (.06)**	0.04 (.04)	0.02 (.04)	−0.03 (.03)	.59**	0.71**	0.03	−1.07**	−0.01
PCD conservation	0.01 (.10)	0.91 (.08)**	−0.08 (.05)	−0.01 (.06)	−0.04 (.04)	.55**	0.93**	−0.13	−0.90**	−0.11
PCD openness	0.31 (.17)	0.81 (.20)**	−0.16 (.07)*	−0.10 (.12)	−0.01 (.05)	.23**	1.11**	−0.28*	−0.50	−0.08
PCD self‐enhancement	0.32 (.15)*	0.26 (.17)	−0.04 (.07)	0.08 (.14)	−0.26 (.06)**	.09**	0.58*	−0.23	0.07	−0.38*
Policy support										
PCD self‐transcendence	−0.34 (.11)**	0.80 (.11)**	0.04 (.06)	−0.05 (.06)	−0.02 (.04)	.36**	0.46**	−0.03	−1.13**	−0.11
PCD conservation	−0.08 (.14)	0.79 (.11)**	−0.14 (.07)	−0.07 (.08)	−0.05 (.05)	.31**	0.71**	−0.25	−0.87**	−0.11
PCD openness	−0.05 (.21)	0.67 (.25)**	−0.13 (.08)	−0.14 (.14)	−0.08 (0.6)	.09**	0.62	−0.34*	−0.71*	−0.07
PCD self‐enhancement	0.24 (.17)	0.09 (.20)	−0.03 (.09)	−0.04 (.16)	−0.27 (.07)**	.06**	0.33	−0.33	0.15	−0.26
Tolerance										
PCD self‐transcendence	−0.14 (.10)	1.03 (.08)**	−0.02 (.05)	−0.06 (.05)	−0.06 (.04)	.49**	0.89**	−0.14	−1.16**	−0.02
PCD conservation	0.18 (.12)	1.08 (.10)**	−0.23 (.07)**	−0.11 (.07)	−0.08 (.04)	.48**	1.26**	−0.42**	−0.90**	−0.20*
PCD openness	0.51 (.20)*	0.96 (.24)**	−0.23 (.08)**	−0.22 (.14)	−0.08 (.06)	.17**	1.47**	−0.53**	−0.46	−0.08
PCD self‐enhancement	0.59 (.17)**	0.31 (.20)	−0.12 (.09)	−0.07 (.17)	−0.30 (.07)**	.10**	0.89**	−0.50**	0.28	−0.35

Note

NL is an abbreviation for the Netherlands and indicates the perception of Dutch host‐society’s values. M is an abbreviation for migrant and indicates the perception of migrant values. The significance of the slopes and response surface coefficients is determined by error estimates; hence, some larger coefficients may not be significant.

* *p* < .05

** *p* < .01. See Appendix S1 for the exact *p*‐values.

**Table 8:** Study 2: Polynomial regression of migrant attitudes by PCD in values in the Polish migrant condition

	Slopes *b* (*SE*)					$R_{\text{adj}}^{2}$	Response Surface Test			
	b_1_	b_2_	b_3_	b_4_	b_5_		a_1_	a_2_	a_3_	a_4_
Attitudes	NL	M	NL^2^	NLM	M^2^					
PCD self‐transcendence	0.09 (.10)	0.60 (.10)**	−0.01 (.05)	−0.07 (.07)	−0.02 (.04)	.29**	0.69**	−0.09**	−0.52**	0.04
PCD conservation	0.06 (.11)	0.51 (.09)**	−0.09 (.07)	0.004 (.07)	−0.03 (.04)	.26**	0.57**	−0.12	−0.45**	−0.13
PCD openness	0.46 (.16)**	−0.03 (.18)	−0.23 (.07)**	0.29 (.11)*	−0.08 (.05)	.17**	0.44**	−0.02	0.49	−0.60**
PCD self‐enhancement	0.25 (.13)*	0.33 (.10)**	−0.12 (.07)	0.02 (.08)	−0.09 (.05)	.08**	0.58**	−0.19**	−0.07	−0.22
Policy support										
PCD self‐transcendence	−0.13 (.13)	0.48 (.13)**	−0.02 (.07)	0.06 (.09)	−0.03 (.06)	.18**	0.36*	0.02	−0.61**	0.05
PCD conservation	0.07 (.15)	0.44 (.11)**	−0.09 (.08)	0.12 (.09)	−0.09 (.05)	.21**	0.51**	−0.06	−0.36	−0.30
PCD openness	−0.37 (.21)	0.28 (0.23)	0.05 (.09)	0.07 (.15)	−0.05 (.06)	.05**	−0.09	0.07	−0.65	−0.07
PCD self‐enhancement	−0.01 (.16)	−0.00 (.12)	0.03 (.09)	0.21 (.10)*	−0.17 (.06)**	.04*	−0.01	0.07	−0.01	−0.34
Tolerance										
PCD self‐transcendence	−0.06 (.12)	0.73 (.13)**	0.10 (.06)	−0.01 (.09)	−0.10 (.06)	.33**	0.67**	−0.01	−0.79**	0.01
PCD conservation	0.01 (.14)	0.60 (.11)**	−0.09 (.08)	0.12 (.09)	−0.10 (.05)*	.31**	0.61**	−0.07	−0.59**	−0.31
PCD openness	0.10 (.20)	0.45 (.23)*	−0.01 (.09)	0.14 (.14)	−0.14 (.06)*	.20**	0.55**	−0.01	−0.35	−0.28
PCD self‐enhancement	0.07 (.16)	0.49 (.13)**	−0.10 (.09)	0.15 (.10)	−0.13 (.06)*	.12**	0.56**	−0.08	−0.41*	−0.38*

Note

NL is an abbreviation for the Netherlands and indicates the perception of Dutch host‐society’s values. M is an abbreviation for migrant and indicates the perception of migrant values. The significance of the slopes and response surface coefficients is determined by error estimates; hence, some larger coefficients may not be significant.

* *p* < .05

** *p* < .01. See Appendix S1 for the exact *p*‐values.

#### PCD in social values

We first tested whether greater PCD in social values was associated with more negative attitudes towards migrants (*Hypothesis 1*). First, both social values explained a considerable proportion of variance in attitudes towards migrants, policy support, and tolerance towards migrants (between 27% and 23%). This was consistent across migrant conditions. Second, for both social values, only the simple slopes for perceived migrant’s values tended to be significant (between *b*_2_ = 0.44 and *b*_2_ = 1.08, *p*’s < .01, with a few exceptions) meaning that the relationship between PCD in social values and migrant attitudes could mostly be explained by the perceived value endorsement of migrants and not by the endorsement of the host‐society.

**Figure 3:**
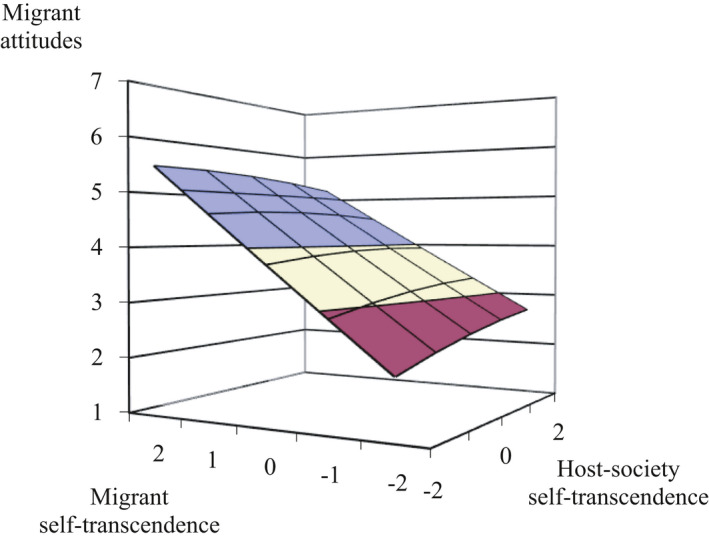
Response surface of PCD in self‐transcendence in the Moroccan migrant condition of Study 2. *Note*. PCD is an abbreviation of perceived cultural distance. Colour in the graph is only intended to improve the graph’s three‐dimensional perspective (the current graph has more colour compared to the previous due to a larger spread). The lines of congruence (*a*_1_) and incongruence (*a*_3_) are steep and linear (also see Figure 1) and the overall graph tends to be straight rather than curved indicating that the relationship between PCD and migrant attitudes is linear (there is a slight curve based on the *a*_2_ and *a*_4_ coefficients, suggesting non‐linearity, but these were non‐significant which is not taken into account when plotting the graph).

Third, most importantly, the line of incongruence (*a*_3_) in the RSA was consistently significant across migrant conditions and types of attitudes indicating a dissimilarity effect (between *a*_3_ = −0.44 and *a*_3_ = −1.16, *p*’s < .01, except in the Polish condition the link between PCD in conversation values and policy support was non‐significant but showing the same pattern). The relationship between PCD in social values and migrant attitudes was clearly linear (see Figure 3 and Appendix S1), meaning that the direction of the dissimilarity mattered. When migrants were perceived to endorse social values *more* than the host‐society, they were evaluated *less* negatively. Moreover, it did not matter to what extent people perceived the society to endorse social values, they prefer migrants to do so anyway. Overall, these results confirmed that when people perceived migrants to endorse social values less than the host‐society, they were more negative towards migrants (*Hypothesis 1*).

#### Comparison of PCD in social and personal values

Next, to test whether PCD in social values was more strongly associated with migrant attitudes than PCD in personal values (*Hypothesis 2*), we investigated the results for personal values. First, PCD in personal values explained less variance in migrant attitudes (between 4% and 23%) compared to the PCD in social values (between 14% and 59%). Second, although some of the slopes and RSA coefficients of the personal values were significant, they were weaker and less consistent than those for social values. Moreover, the relationship between PCD in personal values and migrant attitudes tended to depend on people’s perceptions of the host‐society (due to simple slopes of host‐society value endorsement, *a*_2_ and *a*_4_ being significant). For example, people who evaluated the host‐society as low in self‐enhancement values supported migrant policies for Moroccan migrants more, if they perceived Moroccan migrants as also being low in endorsing self‐enhancement. This single finding only emerged in the Moroccan condition for the link between self‐enhancement and policy support. Although PCD in the personal values was linked to some aspects of migrant attitudes, the effect sizes (*R* ^2^ and slopes) were much smaller and results more inconsistent than those for PCD in social values. Altogether, and in line with Study 1, these results confirmed *Hypothesis 2*: PCD in social values was more strongly associated with migrant attitudes than PCD in personal values.

### Mediation by symbolic threat

We used Hayes’s (2013) PROCESS software (Model 4, bootstrapping with 5,000 resamples) to test whether symbolic threat would mediate the relation between PCD in social values and migrant attitudes^2^. To our knowledge, this cannot be done in SPSS with polynomial statistics. Hence, we subtracted perceived migrant values from the perceived Dutch values and used this difference score. We ran this analysis for each migrant condition, for all types of PCD in values, and with the general attitudes towards migrants as dependent variable. Across all migrant conditions, the mediation by threat was significant for PCD in social values, confirming *Hypothesis 3* (see Table 9)^3^. Regarding personal values, only one mediation analysis was significant, namely for PCD in openness in the Syrian condition.

**Table 9:** Study 2: Mediation by symbolic threat per migrant condition

	Indirect effect (95% CI)	PCD to ST	ST to MA	PCD to MA (PCD to MA with ST)
Moroccan migrants				
PCD self‐transcendence	−0.17 (−0.26, −0.08)*	0.46**	−0.25**	−0.47** (−0.36**)
PCD conservation	−0.17 (−0.27, −0.08)*	0.54**	−0.32**	−0.46** (−0.28**)
PCD openness	−0.11 (−0.22, −0.01)*, †	0.25**	−0.43**	−0.29** (−0.18**)
PCD self‐enhancement	0.08 (−0.02, 0.18)	−0.18*	−0.47**	−0.09 (−.17**)
Syrian migrants				
PCD self‐transcendence	−0.23 (−0.32, −0.15)*	0.62**	−0.36**	−0.54** (−0.32**)
PCD conservation	−0.21 (−0.29, −0.14)*	0.58**	−0.37**	−0.53** (−0.32**)
PCD openness	−0.19 (−0.30, −0.09)*	0.39**	−0.49**	−0.41** (−0.22**)
PCD self‐enhancement	0.14 (0.01, 0.27)*, †	−0.25**	−0.54**	0.07 (−0.07)
Polish migrants				
PCD self‐transcendence	−0.14 (−0.23, −0.06)*	0.54**	−0.25**	−0.27** (−0.14*)
PCD conservation	−0.11 (−0.19, −0.04)*	0.52**	−0.21**	−0.34** (−0.23**)
PCD openness	−0.07 (−0.15, 0.01)	0.24*	−0.28**	−0.23** (−0.16*)
PCD self‐enhancement	−0.04 (−0.12, 0.03)	0.12	−0.29**	−0.09 (−0.05)

Note

The mediation effect is significant when the confidence interval (CI) does not include zero. PCD is an abbreviation for perceived cultural distance, ST for symbolic threat, and MA for migrant attitudes.

* *p* < .05

** *p* < .01

† No longer significant after controlling for age, gender, education, and political orientation.

### Internal meta‐analyses

**Figure 4:**
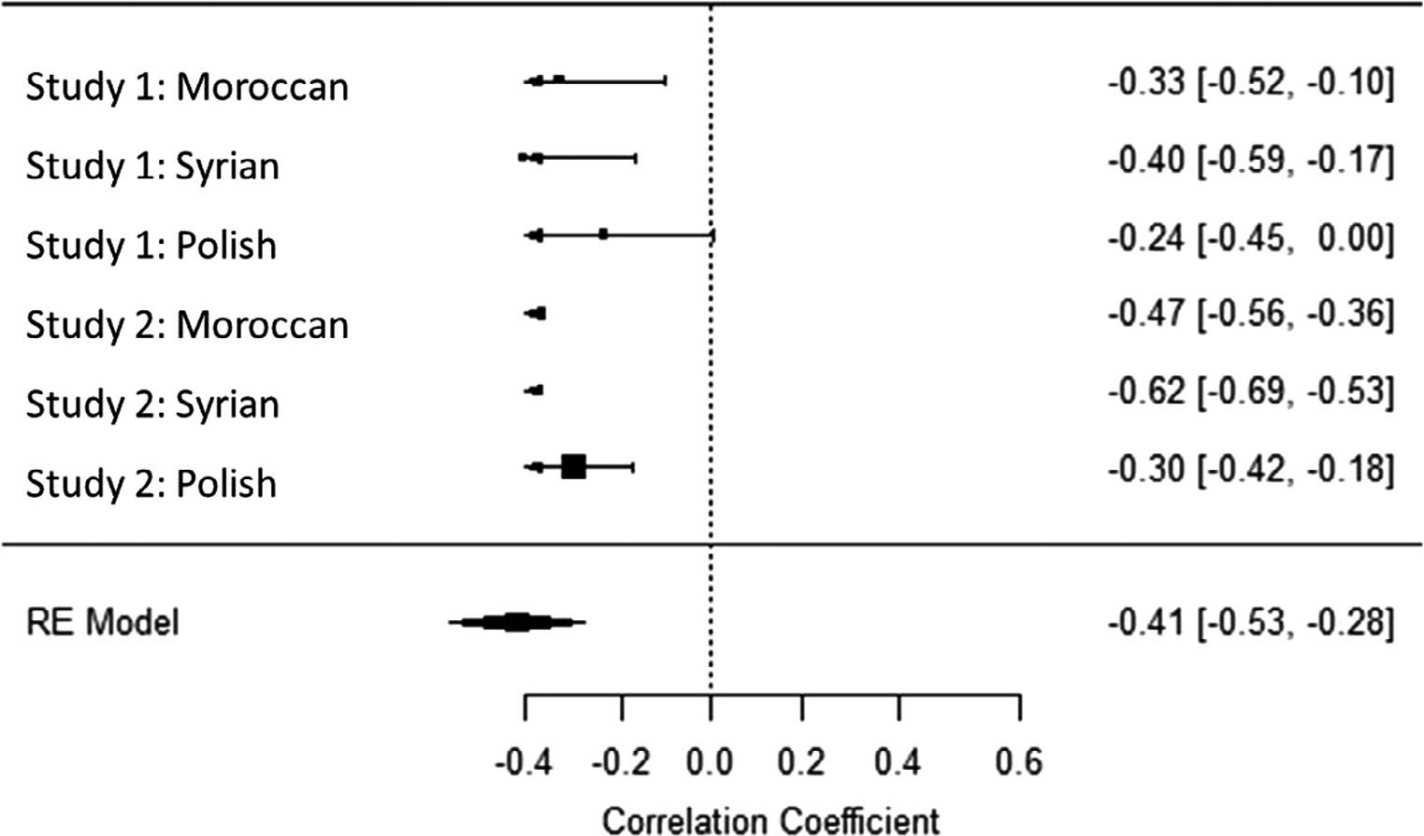
Forest plot of the meta‐analysis of perceived cultural distance in self‐transcendence and migrant attitudes.

**Figure 5:**
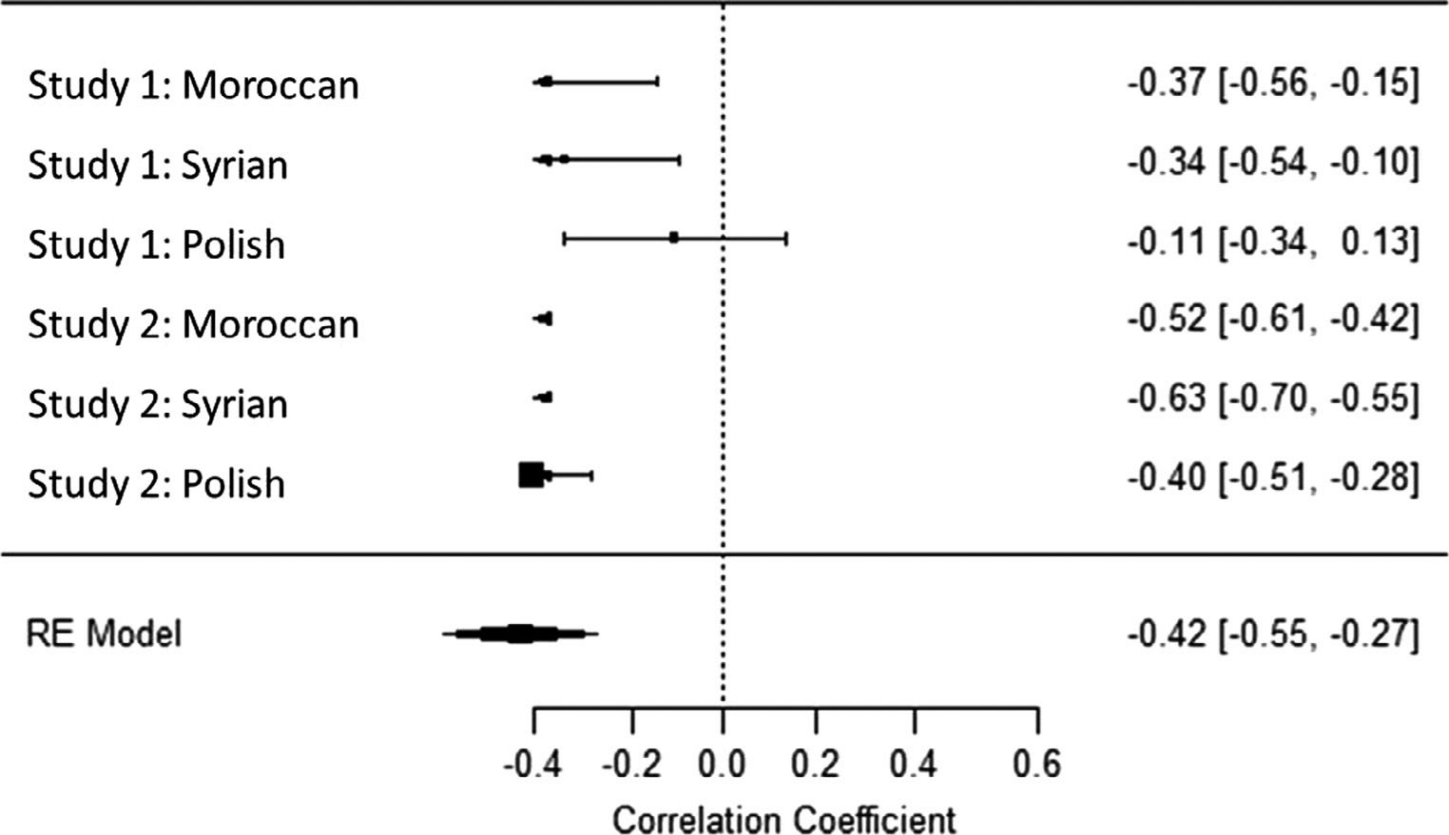
Forest plot of the meta‐analysis of perceived cultural distance in conservation and migrant attitude.

**Figure 6:**
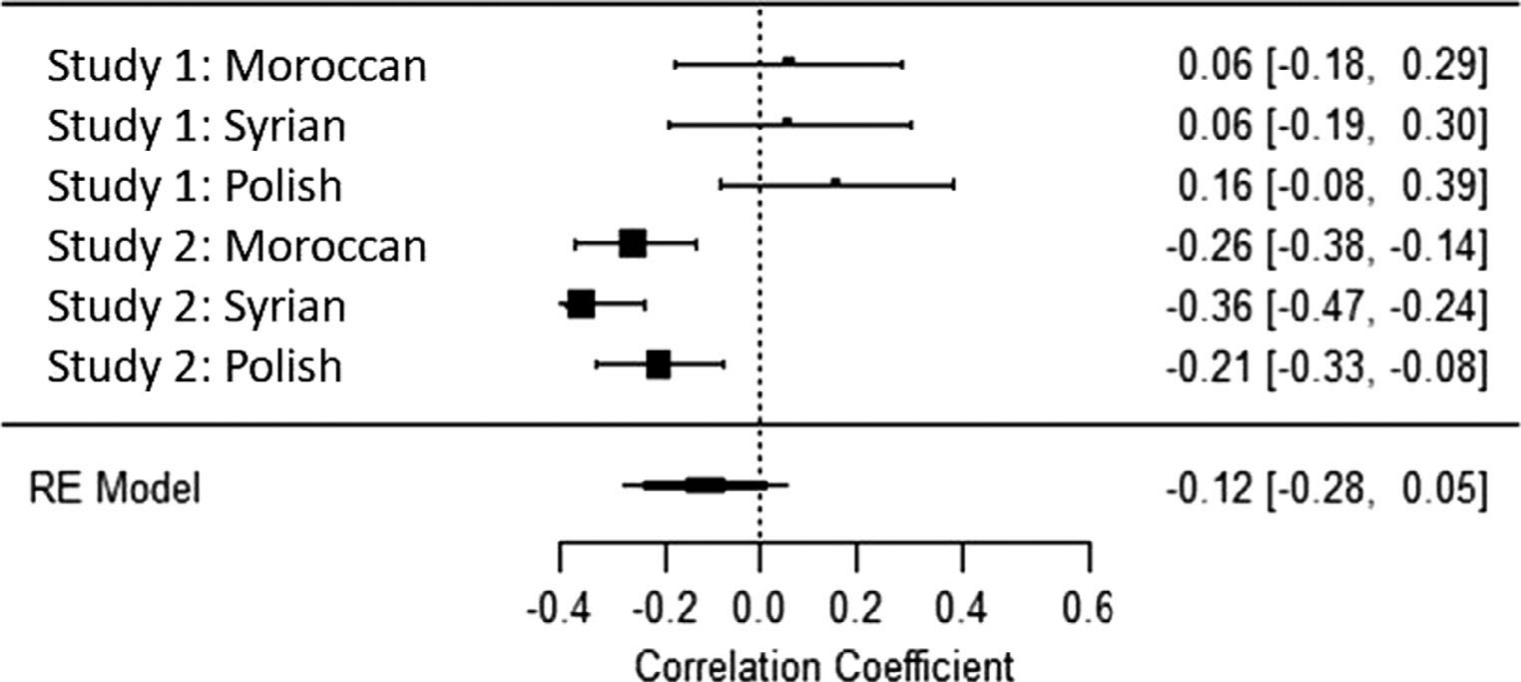
Forest plot of the meta‐analysis of perceived cultural distance in openness and migrant attitude.

**Figure 7:**
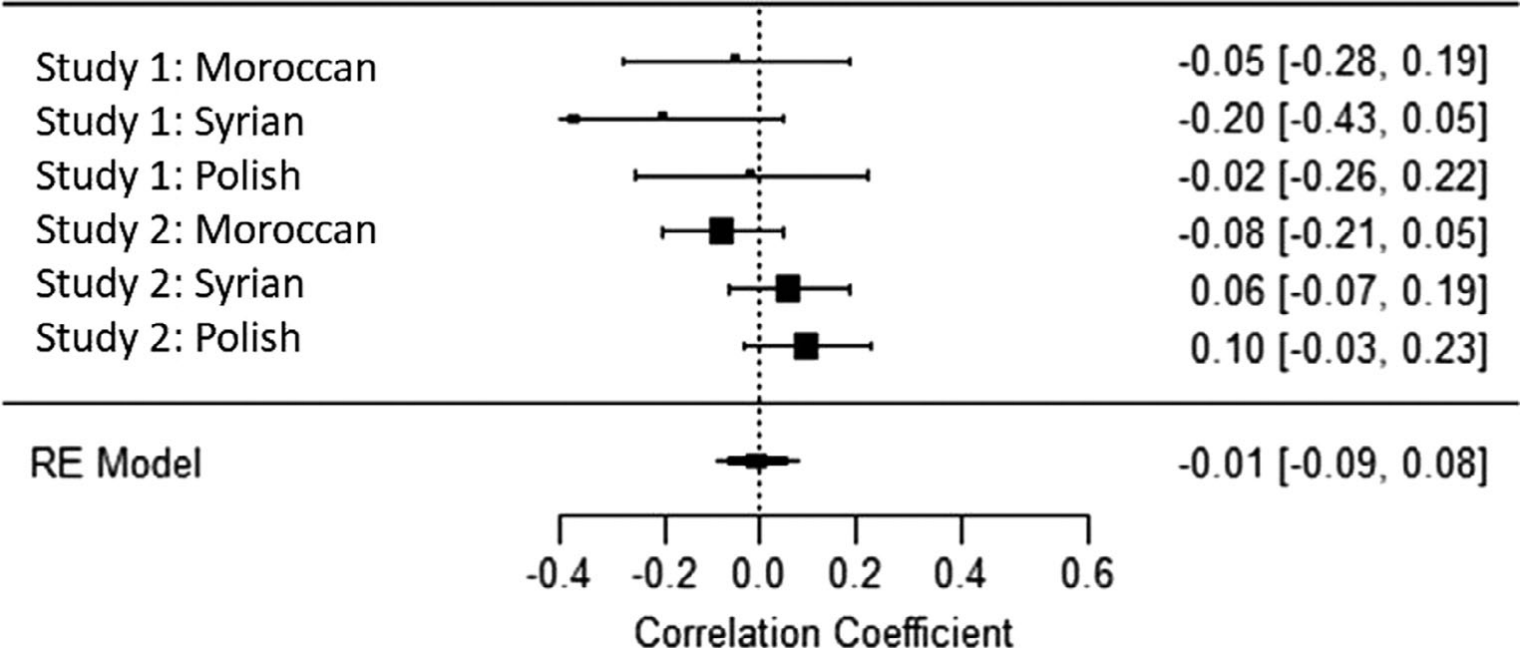
Forest plot of the meta‐analysis of perceived cultural distance in self‐enhancement and migrant attitude.

To comprehensively summarize our findings and make interferences and comparisons of their effect sizes, we conducted four internal mini‐meta‐analyses for the correlations of the four PCDs in values with migrant attitudes across the two studies and the three migrant conditions. To the best of our knowledge, meta‐analyses cannot be conducted with polynomial regression statistics; thus, we again used a difference‐score for PCD in values. Analyses were computed in R, package *metafor* (R Development Core Team, 2010; Viechtbauer, 2010). We used random effects (maximum likelihood) in which the mean effect size was weighted by sample size. All correlations were Fischer’s *z* transformed and converted back to Pearson correlation for ease of interpretation. The mean effect size of the relationship between PCD in social values and migrant attitudes was *r* = −.41 for self‐transcendence and *r* = −.42 for conservation (both *p’*s < .01, see Figures 4, 5, 6, 7). The mean effect size for the PCD in personal values was *r* = −.12, *p* = 0.18, for openness, and *r* = −.01, *p* = .86, for self‐enhancement. These results confirmed that PCD in social values, but not personal values, was systematically associated with migrant attitudes across studies and conditions; the effect sizes were moderate to large. When analysing PCD in all four different values in one regression analysis, this further confirms our conclusions (see Appendix S1 for details).

## GENERAL DISCUSSION

This research investigated *when* and *why* PCD is associated with negative attitudes towards migrants. Novel to previous research on PCD, we specifically investigated PCD in cultural values and differentiated between PCD in social values—focussing on others’ welfare (self‐transcendence), and how to behave in society (conservation)—and PCD in personal values—focussing on personal needs and gains (openness and self‐enhancement). Our findings confirmed that when host‐society members perceived migrants to endorse social values *less* than the host‐society, this was associated with more negative attitudes towards migrants, less support for policies improving migrants’ position in society, and less tolerance towards migrants.

Due to the use of new advanced analyses (i.e., polynomial regression and response surface analyses), we could conclude that this relationship was linear; meaning that the direction of PCD in social values mattered. When migrants were perceived to endorse social values *more* than the host‐society, people had *less* negative attitudes towards migrants. Hence, the host‐society seemed to accept cultural distance towards migrants if they perceived them to endorse social values more, but not less, strongly. Furthermore, this relationship was relatively independent of the degree people perceived their host‐society to endorse social values. Even when assuming that the host‐society did not endorse social values, they preferred migrants to do so, nonetheless. Importantly, these results systematically replicated across studies, different types of migrant attitudes (general attitudes, policy support, tolerance), and across migrant groups of different origin (Moroccan, Syrian, Polish). Moreover, an internal meta‐analysis across the studies and migrant groups revealed that the relationship between PCD in social values and migrant attitudes was moderate to large.

As expected, our data suggest that the link between PCD in social values and attitudes towards migrants was mediated by symbolic threat. Although previous research already demonstrated that when outgroups are perceived as incompatible with the ingroup, this is associated with negative outgroup attitudes (Riek et al., 2006), our research adds insights into *which* differences may be considered especially incompatible and, accordingly, threatening. Social values play an important role in the functioning of groups and their social interactions (Schwartz & Bardi, 2001). When people interact or coexist in society, social values are important guidelines for social behaviour. People may be especially sensitive to (objective or subjective) information regarding social values of members of other groups, as their endorsement of these values may directly affect their interactions. Hence, when migrant groups are believed to endorse social values to a lesser extent than the host‐society, this will likely influence intergroup relations negatively.

As hypothesized, and consistent across studies, types of migrant attitudes, and migrant groups, PCD in social values was more strongly associated with migrant attitudes than PCD in personal values. Interestingly, some personal values (e.g., self‐direction and stimulation) were considered especially important in Dutch society (Schwartz, 2006), and people perceived migrant groups to differ substantially in their endorsement of these values. Yet, different from social values, the associations between PCD in personal values and migrant attitudes varied strongly across migrant groups and measures for capturing migrant attitudes. Moreover, PCD on specific value dimensions was more strongly linked to migrant attitudes than the general PCD, supporting our idea that it is relevant to zoom in on specific dissimilarities between host‐society and migrant groups to better understand their intergroup relation. Altogether, these results suggest that host‐society members may be able to accept PCD regarding some aspects (e.g., personal values), but are intolerant of cultural outgroups that are perceived as rejecting or only weakly endorsing social values. Still, we cannot exclude the possibility that in other contexts than addressed in the present research, personal values may play a larger role. For example, for migrants having close interpersonal relations with host‐society members, PCD in personal values may be more noticeable and relevant compared to the broader intergroup context in the present research.

In line with research on the concordance model of acculturation (Piontkowski et al., 2002), social values are related to expectations about acculturation orientations. Acculturation strategies refer to the varying pathways newcomers might take when adapting to a new or unfamiliar culture. They can choose to maintain their heritage culture or to have contact and participate meaningfully with the host‐society (and forms in between; Berry, 1992). Mismatching expectations about acculturation (e.g., a host‐society expecting assimilation while a migrant group prefers separation) would entail different ways of living together or apart in one society, which relate to social values.

Other research showed that not all cultural practices of minority groups are rejected to the same extent (e.g., Adelman & Verkuyten, 2020; Van der Noll, 2014). *Which* cultural practices are rejected may be determined by the associated perceived differences in underlying social values. For example, in the Netherlands the opposition against the religious wearing of headscarves may not be grounded in the particular practice itself, but in the value perceived to be violated: people oppose headscarves because they consider them a symbol of gender inequality, thereby violating the social value of universalism. Accordingly, when differences in cultural practices give rise to intergroup tension, the associated underlying values of the respective practices may offer valuable insights.

### Limitations and future directions

There are three main limitations of our research. First, both studies had a cross‐sectional design. Accordingly, we cannot draw causal conclusions based on our data. The question remains whether PCD in social values increases anti‐migrant attitudes or whether people holding anti‐migrant attitudes, subsequently see migrants as disregarding social values (i.e., a process of dehumanization, e.g., Greenhalgh & Watt, 2015). Another possibility is that PCD in social values and attitudes mutually influence each other. An interesting venue for future research could be to examine whether PCD in social values can be reduced, and whether this may decrease threat and prejudice. For now, we can only state that the reversed mediation model explained less variance compared to our proposed model.

Second, we conducted our research among Dutch host‐society members; we cannot exclude the possibility that the findings are specific to this context. There are, however, findings suggesting that the importance of PCD in social values is a stable phenomenon. Cross‐cultural research on values in 56 different nations showed strong consistency in the hierarchy of values and the importance of social values (Fischer & Schwartz, 2011; Schwartz & Bardi, 2001). Given that the structure of values seems universal, future research should test whether the association between PCD in social values and migrant attitudes is consistent across different cultures.

Finally, our findings regarding the Polish migrant group were not fully consistent. In Study 1, the link between PCD in social values and attitudes towards Polish migrants was only marginally significant. However, the subsamples were relatively small, weakening the statistical power of the analysis. Study 2, with a larger and more representative sample, demonstrated that the link between PCD in social values and migrant attitudes also applied to Polish migrants.

### Conclusions

To conclude, when members of the host‐society perceive larger cultural distance towards migrants, this is associated with more negative outgroup attitudes (Mahfud et al., 2018). Yet, cultural differences between groups are not problematic per se (Adelman & Verkuyten, 2020; Jetten et al., 2004). After all, people want their ingroup to be distinct from outgroups (Tajfel & Turner, 1979), and they are able and willing to tolerate some cultural differences (Verkuyten, 2018). Indeed, our studies reveal that host‐society members can perceive cultural distance towards migrant groups without this necessarily being associated with negative attitudes towards migrants. Importantly, especially PCD in social values may mark an important turning point for intercultural group relations, as it was, via its association with symbolic threat, consistently linked to more negative evaluations of migrants. Hopefully, our findings will inspire future research to broaden our understanding of *how* to spark more intergroup harmony and may help developing interventions to create more inclusive societies.

## Conflicts of interest

The authors declared that they had no conflict of interests with respect to their authorship or the publication of this article.

## Author contributions

Katja Albada (Conceptualization; Formal analysis; Funding acquisition; Methodology; Project administration; Visualization; Writing – original draft; Writing – review & editing) Nina Hansen (Conceptualization; Funding acquisition; Methodology; Writing – original draft; Writing – review & editing) Sabine Otten (Conceptualization; Methodology; Writing – original draft; Writing – review & editing)

## Ethical considerations

Each study was approved in advance by the Ethics Committee of the Department of Psychology at the University of Groningen and was in accordance with the Declaration of Helsinki, the National Ethics Council of Social and Behavioral Sciences, and the APA.

## Supporting information


**Appendix S1** Additional statistics, analyses, and figures.

## Data Availability

The data that support the findings of this study are openly available on the Open Science Framework at: https://osf.io/dr8vu/

## Footnotes

^1^
There is no formal legal definition of an international migrant. It is an umbrella term for someone who has changed his/her country of usual residence. For ease of reading, we use the term migrant group and specify the immigration origin when referring to the three distinct groups in the *Current Research* section.

^2^
Testing the mediation in Study 1 revealed fully comparable results (see Supporting Information); for word limitations, we only reported results of Study 2.

^3^
The reversed mediation models explained less variance than our proposed models (see Supporting Information).
