# Supplementary material for: When cultures clash: Links between perceived cultural distance in values and attitudes towards migrants
Source: Br J Soc Psychol. 2021 May 9;60(4):1350–78. doi: 10.1111/bjso.12455 (PMC8518719; doi:10.1111/bjso.12455)
Supplement: Supplementary file 1 — Appendix S1 Additional statistics, analyses, and figures. [file BJSO-60-1350-s001.docx]

**Supporting Information**

**Table 1.** Study 1: Cronbach’s Alphas per migrant condition.

|  | **Moroccan migrants** | | | **Syrian migrants** | | **Polish migrants** | |
| --- | --- | --- | --- | --- | --- | --- | --- |
|  | | NL | Migrant | NL | Migrant | NL | Migrant |
| Self-transcendence | | .74 | .76 | .70 | .75 | .74 | .79 |
| Conservation | | .72 | .80 | .55 | .79 | .64 | .76 |
| Openness | | .69 | .85 | .68 | .87 | .78 | .81 |
| Self-enhancement | | .77 | .80 | .80 | .81 | .79 | .86 |
| Migrant attitudes | | .92 | | .85 | | .74 | |

**Symbolic threat mediation in Study 1**

In Study 1, we also tested whether symbolic threat would mediate the relationship between PCD in social values and attitudes towards migrants (*Hypothesis 3*). However, we decided to only report the results from Study 2 in the manuscript as they yielded similar results and in order to stay within the word limit. We decided to report the results of Study 2 in the manuscript because the sample size allowed for separate analyses whereas the sample size of Study 1 was too small to reliability run the analysis in each migrant condition separately.

In Study 1, the scale of symbolic threat in the overall sample was reliable with a Cronbach’s alpha of .75 (.79, .73, and.70 in the Moroccan, Syrian, and Polish migrant conditions respectively). See Table 2 for the means, standard deviations, and correlations. We conducted the mediation analysis in the overall sample (due to the sample size) for PCD in all four types of values. We used Hayes’s (2013) PROCESS software (Model 4, bootstrapping with 5,000 resamples) to test the mediation. We used the bootstrapped confidence intervals to determine whether the indirect effect was significant (Hayes & Scharkow, 2013). The indirect effect is significant when the confidence interval does not include zero. When the indirect effect is significant, mediation has occurred. The mediation by symbolic threat was significant for PCD in both social values (see Table 3). These analyses provided support for our mediation hypothesis (*Hypothesis 3*).

**Table 2.** Study 1: Descriptive statistics and Pearson’s correlations for symbolic threat.

| **Migrant conditions** | | **Correlations with symbolic threat** | | | | |
| --- | --- | --- | --- | --- | --- | --- |
|  | *M* (*SD*) | PCD self- trans | PCD conserve | PCD openness | PCD self-enhance | Migrant attitudes |
| Moroccan | 3.64 (1.34) | .48** | .39** | .23 | -.15 | -.43** |
| Syrian | 3.12 (1.11) | .45** | .32* | .12 | -.14 | -.58** |
| Polish | 2.96 (0.97) | .06 | .15 | .08 | .10 | -.23^†^ |

*Note*. Based on an one-way ANOVA [*F(*2,198) = 6.36, *η ^2^*= .06, *p* < .01] Moroccan migrants were evaluated as significantly more threatening compared to Syrian and Polish migrants (*p* < .05). Self-trans is an abbreviation for self-transcendence, conserve for conservation, and self-enhance for self-enhancement. Lower migrant attitudes indicate more negative attitudes. **p* < .05. ***p* < .01. ^†^*p* = .063.

**Table 3.** Study 1: Mediation by symbolic threat in the overall sample.

|  | Indirect effect  (95% *CI*) | PCD to ST | ST to MA | PCD to MA (PCD to Ma with ST) |
| --- | --- | --- | --- | --- |
| PCD self-transcendence | -0.08 (-0.13, -0.04)* | 0.40** | -0.20** | -0.27** (-0.19**) |
| PCD conservation | -0.06 (-0.11, -0.02)* | 0.29** | -0.21** | -0.20** (-0.14*) |
| PCD openness | -0.05 (-0.11, -0.01)* | -0.22** | -0.25** | 0.06 (0.00) |
| PCD self-enhancement | -0.03 (-0.01, 0.07) | 0.11 | -0.24** | -0.07 (-0.04) |

*Note.* The mediation effect is significant when the confidence interval (*CI*) does not include zero. PCD is an abbreviation for perceived cultural distance, ST for symbolic threat, and MA for migrant attitudes. **p* < .05. ** *p* < .01.

**Table 4.** Study 1: *P*-values of the polynomial regression of attitudes by PCD in values.

|  | **Slopes *b*** | | | | |  | **Response Surface Test** | | | |
| --- | --- | --- | --- | --- | --- | --- | --- | --- | --- | --- |
|  | **b_1_**  *p* | **b_2_**  *p* | **b_3_**  *p* | **b_4_**  *p* | **b_5_**  *p* | **R_adj_^2^**  *p* | **a_1_**  *p* | **a_2_**  *p* | **a_3_**  *p* | **a_4_**  *p* |
| **Overall sample** | NL | M | NL^2^ | NLM | M^2^ |  |  |  |  |  |
| Self-transcendence | .950 | .007 | .944 | .881 | .409 | .000 | .110 | .626 | .029 | .694 |
| Conservation | .715 | .000 | .217 | .726 | .586 | .000 | .003 | .474 | .001 | .184 |
| Openness | .694 | .270 | .392 | .551 | .197 | .048 | .723 | .698 | .309 | .180 |
| Self-enhancement | .905 | .377 | .313 | .897 | .588 | .540 | .572 | .431 | .538 | .366 |
| **Moroccan migrants** |  |  |  |  |  |  |  |  |  |  |
| Self-transcendence | .921 | .116 | .944 | .727 | .877 | .001 | .376 | .715 | .121 | .772 |
| Conservation | .772 | .016 | .311 | .418 | .689 | .000 | .154 | .904 | .019 | .269 |
| Openness | .083 | .433 | .062 | .545 | .490 | .268 | .210 | .212 | .051 | .032 |
| Self-enhancement | .033 | .089 | .152 | .036 | .412 | .319 | .031 | .049 | .747 | .432 |
| **Syrian migrants** |  |  |  |  |  |  |  |  |  |  |
| Self-transcendence | .882 | .194 | .712 | .737 | .795 | .005 | .463 | .900 | .220 | .745 |
| Conservation | .337 | .013 | .328 | .438 | .147 | .019 | .215 | .392 | .012 | .101 |
| Openness | .326 | .895 | .200 | .933 | .591 | .513 | .486 | .487 | .333 | .512 |
| Self-enhancement | .732 | .977 | .613 | .106 | .079 | .177 | .816 | .889 | .804 | .035 |
| **Polish migrants** |  |  |  |  |  |  |  |  |  |  |
| Self-transcendence | .847 | .597 | .900 | .696 | .112 | .000 | .712 | .265 | .584 | .318 |
| Conservation | .084 | .059 | .530 | .921 | .828 | .010 | .023 | .902 | .592 | .726 |
| Openness | .269 | .447 | .361 | .658 | .007 | .004 | .175 | .906 | .895 | .427 |
| Self-enhancement | .952 | .889 | .363 | .832 | .300 | .454 | .974 | .237 | .889 | .306 |

*Note.* NL is an abbreviation for the Netherlands and indicates the evaluation of Dutch host-society’s values. M is an abbreviation for migrant and indicates the evaluation of migrant values.

**Table 5.** Study 2: Cronbach’s Alphas per migrant condition.

|  | **Moroccan migrants** | | **Syrian migrants** | | **Polish migrants** | |
| --- | --- | --- | --- | --- | --- | --- |
|  | NL | Migrant | NL | Migrant | NL | Migrant |
| Self-transcendence | .88 | .86 | .89 | .90 | .89 | .90 |
| Conservation | .84 | .82 | .81 | .88 | .83 | .89 |
| Openness | .87 | .84 | .84 | .86 | .88 | .90 |
| Self-enhancement | .79 | .81 | .82 | .80 | .82 | .83 |
| Symbolic threat | .84 | | .87 | | .83 | |
| Migrant attitudes | .96 | | .97 | | .94 | |
| Policy support | .67 | | .73 | | .55 | |
| Tolerance | .85 | | .89 | | .85 | |

**Table 6.** Study 2: *P*-values of the polynomial regression of migrant attitudes by PCD in values in the Moroccan migrant condition.

|  | **Slopes *b*** | | | | |  | **Response Surface Test** | | | | |
| --- | --- | --- | --- | --- | --- | --- | --- | --- | --- | --- | --- |
|  | **b_1_**  *p* | **b_2_**  *p* | **b_3_**  *p* | **b_4_**  *p* | **b_5_**  *p* | | **R_adj_^2^**  *p* | **a_1_**  *p* | **a_2_**  *p* | **a_3_**  *p* | **a_4_**  *p* |
| **Attitudes** | NL | M | NL^2^ | NLM | M^2^ | |  |  |  |  |  |
| Self-transcendence | .672 | .000 | .718 | .353 | .909 | | .000 | .000 | .432 | .000 | .666 |
| Conservation | .098 | .000 | .465 | .614 | .046 | | .000 | .000 | .093 | .000 | .318 |
| Openness | .745 | .006 | .578 | .374 | .561 | | .000 | .155 | .581 | .028 | .461 |
| Self-enhancement | .434 | .004 | .677 | .115 | .001 | | .001 | .242 | .698 | .022 | .025 |
| **Policy support** |  |  |  |  |  | |  |  |  |  |  |
| Self-transcendence | .415 | .000 | .138 | .785 | .984 | | .000 | .001 | .526 | .003 | .336 |
| Conservation | .088 | .000 | .165 | .131 | .149 | | .000 | .028 | .619 | .000 | .030 |
| Openness | .701 | .884 | .277 | .188 | .477 | | .010 | .718 | .978 | .829 | .083 |
| Self-enhancement | .578 | .396 | .149 | .011 | .027 | | .006 | .326 | .554 | .939 | .000 |
| **Tolerance** |  |  |  |  |  | |  |  |  |  |  |
| Self-transcendence | .298 | .000 | .799 | .257 | .838 | | .000 | .000 | .376 | .000 | .461 |
| Conservation | .081 | .000 | .690 | .816 | .682 | | .000 | .018 | .887 | .000 | .857 |
| Openness | .216 | .019 | .260 | .923 | .116 | | .000 | .022 | .135 | .521 | .267 |
| Self-enhancement | .333 | .002 | .424 | .383 | .002 | | .003 | .221 | .702 | .009 | .168 |

*Note*. NL is an abbreviation for the Netherlands and indicates the evaluation of Dutch host-society’s values. M is an abbreviation for migrant and indicates the evaluation of migrant values.

**Table 7.** Study 2: *P*-values of the polynomial regression of migrant attitudes by PCD in values in the Syrian migrant condition.

|  | **Slopes *b*** | | | |  | | | **Response Surface Test** | | | |
| --- | --- | --- | --- | --- | --- | --- | --- | --- | --- | --- | --- |
|  | **b_1_**  *p* | **b_2_**  *p* | **b_3_**  *p* | **b_4_**  *p* | | **b_5_**  *p* | **R_adj_^2^**  *p* | **a_1_**  *p* | **a_2_**  *p* | **a_3_**  *p* | **a_4_**  *p* |
| **Attitudes** | NL | M | NL^2^ | NL*M | | M^2^ |  |  |  |  |  |
| PCD self-transcendence | .014 | .000 | .295 | .614 | | .294 | .000 | .000 | .647 | .000 | .853 |
| PCD conservation | .892 | .000 | .133 | .883 | | .289 | .000 | .000 | .144 | .000 | .210 |
| PCD openness | .067 | .000 | .017 | .406 | | .784 | .000 | .000 | .034 | .054 | .606 |
| PCD self-enhancement | .030 | .138 | .608 | .600 | | .000 | .000 | .018 | .143 | .757 | .048 |
| **Policy support** |  |  |  |  | |  |  |  |  |  |  |
| PCD self-transcendence | .002 | .000 | .506 | .376 | | .661 | .000 | .003 | .723 | .000 | .104 |
| PCD conservation | .557 | .000 | .066 | .397 | | .377 | .000 | .001 | .054 | .000 | .238 |
| PCD openness | .825 | .007 | .127 | .348 | | .217 | .000 | .054 | .033 | .027 | .714 |
| PCD self-enhancement | .156 | .658 | 771 | .819 | | .000 | .003 | .240 | .053 | .528 | .244 |
| **Tolerance** |  |  |  |  | |  |  |  |  |  |  |
| PCD self-transcendence | .150 | .000 | .706 | .286 | | .153 | .000 | .000 | .065 | .000 | .770 |
| PCD conservation | .133 | .000 | .001 | .129 | | .081 | .000 | .000 | .001 | .000 | .026 |
| PCD openness | .013 | .000 | .006 | .120 | | .217 | .000 | .000 | .001 | .150 | .662 |
| PCD self-enhancement | .001 | .132 | .153 | .655 | | .000 | .000 | .002 | .004 | .256 | .119 |

*Note*. NL is an abbreviation for the Netherlands and indicates the evaluation of Dutch host-society’s values. M is an abbreviation for Migrant and indicates the evaluation of migrant values.

**Table 8.** Study 2: *P*-values of the polynomial regression of migrant attitudes by PCD in values in the Polish migrant condition.

|  | **Slopes *b*** | | | | |  | **Response Surface Test** | | | |
| --- | --- | --- | --- | --- | --- | --- | --- | --- | --- | --- |
|  | **b_1_**  *p* | **b_2_**  *p* | **b_3_**  *p* | **b_4_**  *p* | **b_5_**  *p* | **R_adj_^2^**  *p* | **a_1_**  *p* | **a_2_**  *p* | **a_3_**  *p* | **a_4_**  *p* |
| **Attitudes** | NL | M | NL^2^ | NLM | M^2^ |  |  |  |  |  |
| PCD self-transcendence | .368 | .000 | .832 | .333 | .733 | .000 | .000 | .005 | .001 | .748 |
| PCD conservation | .590 | .000 | .155 | .953 | .389 | .000 | .000 | .061 | .009 | .311 |
| PCD openness | .004 | .882 | .001 | .010 | .095 | .000 | .004 | .755 | .106 | .002 |
| PCD self-enhancement | .047 | .001 | .098 | .825 | .071 | .001 | .000 | .009 | .645 | .093 |
| **Policy support** |  |  |  |  |  |  |  |  |  |  |
| PCD self-transcendence | .316 | .000 | .801 | .500 | .604 | .000 | .022 | .779 | .004 | .766 |
| PCD conservation | .620 | .000 | .286 | .164 | .054 | .000 | .001 | .456 | .088 | .068 |
| PCD openness | .078 | .225 | .568 | .632 | .411 | .008 | .664 | .365 | .100 | .791 |
| PCD self-enhancement | .942 | .995 | .699 | .032 | .005 | .019 | .948 | .434 | .956 | .045 |
| **Tolerance** |  |  |  |  |  |  |  |  |  |  |
| PCD self-transcendence | .623 | .000 | .125 | .901 | .069 | .000 | .000 | .796 | .000 | .961 |
| PCD conservation | .927 | .000 | .298 | .154 | .030 | .000 | .000 | .411 | .006 | .058 |
| PCD openness | .624 | .048 | .951 | .343 | .025 | .000 | .005 | .848 | .362 | .267 |
| PCD self-enhancement | .653 | .000 | .291 | .121 | .032 | .000 | .007 | .436 | .044 | .028 |

*Note*. NL is an abbreviation for the Netherlands and indicates the evaluation of Dutch host-society’s values. M is an abbreviation for Migrant and indicates the evaluation of migrant values.

**Table 9.** Study 1: Mediation models with PCD in social values.

| **Model** | ***DV*** | ***R*** | ***R^2^*** | ***MSE*** | ***F*** | ***p*** |
| --- | --- | --- | --- | --- | --- | --- |
| **PCD in self-transcendence** | | | | | | |
| 1 | Migrant attitudes | .47 | .22 | 0.43 | 27.22 | <.01 |
| 2 | PCD | .41 | .17 | 0.80 | 19.89 | <.01 |
| **PCD in conservation** | | | | | | |
| 1 | Migrant attitudes | .45 | .21 | 0.43 | 24.94 | <.01 |
| 2 | PCD | .36 | .13 | 1.26 | 14.85 | <.01 |

*Note.* DV is dependent variable. *MSE* is mean squared error. *F*(*df*) = (2,193). Our proposed model in the manuscript (Model 1), predicts more variance in the dependent variable and fits the data better compared to the reversed model (Model 2).

**Table 10.** Study 2: Mediation models with PCD in social values.

| **Model** | ***DV*** | ***R*** | ***R^2^*** | ***MSE*** | ***F*** | ***p*** |
| --- | --- | --- | --- | --- | --- | --- |
| **PCD in self-transcendence** | | | | | | |
| 1 | Migrant attitudes | .61 | .37 | 0.82 | 190.95 | <.01 |
| 2 | PCD | .54 | .29 | 1.17 | 134.02 | <.01 |
| **PCD in conservation** | | | | | | |
| 1 | Migrant attitudes | .63 | .39 | 0.79 | 210.00 | <.01 |
| 2 | PCD | .57 | .33 | 1.19 | 155.59 | <.01 |

*Note.* DV is dependent variable. *MSE* is mean squared error. *F*(*df*) = (2,644). Our proposed model in the manuscript (Model 1), predicts more variance in the dependent variable and fits the data better compared to the reversed model (Model 2).

**Regular Regression Analyses With All Four Types of Values**

For an additional comparison of PCD in the four types of values and their links to attitudes towards migrants, we conducted regular regression analyses with all types of PCD in values in one regression. For these analyses we used difference scores (host-society values minus migrant values). We conducted these analyses for both studies. First, we reported the results for Study 1 (see Table 11). In the Moroccan migrant condition, the regression model was significant with PCD in conservation values as the only significant predictor of attitudes towards migrants [*F*(4,64) = 4.39, *R^2^* = .22, *R*^adj^*^2^* = .17]. In the Syrian migrant condition, the regression model was significant with PCD in self-transcendence as the only significant predictor of attitudes towards migrants [*F*(4,56) = 3.42, *p* = .01, *R^2^* = .20, *R*^adj^*^2^* = .14]. In the Polish migrant condition, the regression model was not significant [*F*(4,61) = 1.75, *p* = .15, *R^2^* = .10, *R*^adj^*^2^* = .04]. However, PCD in self-transcendence was marginally significant (*p* = .09), thus showing a similar pattern in social values as in the other conditions. It is important to note that the PCD in the two social values correlate with each other (see Table 13). When they are both in the analysis, only one may be significant. These analyses suggest that perceiving more cultural distance in social values (self-transcendence or conservation) was associated with more negative attitudes towards migrants (showing support for *Hypothesis 1*). Moreover, PCD in social values was more strongly associated with attitudes towards migrant than PCD in personal values (showing support for *Hypothesis 2*).

Second, we reported the results for Study 2 (see Table 13). Similar to the findings of Study 1, across migrant conditions, PCD in social values (self-transcendence or conservation or both) significantly predicted attitudes towards migrants. This was the case for all three types of migrant attitudes. Hence, when people perceived migrants to endorse social values less than the host-society, they were more negative towards migrants, were less supportive of migrant policies, and tolerated the migrant groups less. These results confirm *Hypothesis 1* and are line with our findings from Study 1. It should be noted that the two PCD in social values highly correlate (see Table 14). When looking at the correlations and the results from the polynomial regression analyses reported in the Manuscript, we can see that PCD in both social values are consistently associated with migrant attitudes. We therefore should not to conclude that one type of social values is more relevant than the other.

Although PCD in the personal values also predicted some aspects of the intergroup relations (e.g., PCD in openness-to-change predicted attitudes towards migrants in the Syrian condition), the effects sizes (*β*’s) were much smaller compared to those for PCD in social values and the results were inconsistent across the migrant groups. These results confirm *Hypothesis 2*; PCD in social values was more strongly associated with migrant attitudes than PCD in personal values. These results are in line with our findings from Study 1. Altogether, these analyses further confirm the pattern that PCD in social values are more strongly linked to migrant attitudes than PCD in personal values.

**Table 11.** Study 1: Regression analysis of PCD in values with migrant attitudes.

|  | **Migrant groups** | | |
| --- | --- | --- | --- |
|  | Moroccan | Syrian | Polish |
|  | *β* | *β* | *β* |
| PCD self-transcendence | -.17 | -.30* | -.26^†^ |
| PCD conservation | -.44** | -.19 | -.13 |
| PCD openness | -.18 | .02 | -.19 |
| PCD self-enhancement | -.23^†^ | .06 | -.12 |
| *R*^adj^*^2^* | .17** | .14* | .04 |

*Note.* PCD is an abbreviation for perceived cultural distance which are difference-scores (host-society values minus migrant values). *β* is the standardized regression coefficient. **p* < .05. ***p* < .01. ^†^*p’s* = .09.

**Table 12.** Study 1: Pearson correlations between PCD in the four types of values.

|  | **Correlations** | | | |
| --- | --- | --- | --- | --- |
|  | 1 | 2 | 3 | 4 |
| 1. PCD self-transcendence | - |  |  |  |
| 2. PCD conservation | .47** | - |  |  |
| 3. PCD openness | .11 | -.25** | - |  |
| 4. PCD self-enhancement | -.39** | -.39** | .01 | - |

*Note.* PCD is an abbreviation for perceived cultural distance which are difference-scores (host-society values minus migrant values). **p* < .05. ***p* < .01. ^†^*p’s* = .09.

**Table 13.** Study 2: Regression analysis of PCD in values with migrant attitudes.

|  | **Type of attitude** | | |
| --- | --- | --- | --- |
|  | Migrant attitudes  *β* | Policy support  *β* | Tolerance  *β* |
| **Moroccan migrants** |  |  |  |
| PCD self-transcendence | -.14 | -.04 | -.06 |
| PCD conservation | -.41** | -.35** | -.29** |
| PCD openness | -.10 | -.13 | -.15* |
| PCD self-enhancement | -.12 | -.01 | -.12 |
| *R*^adj^*^2^* | .30** | .17** | .16** |
| **Syrian migrants** |  |  |  |
| PCD self-transcendence | -.25* | -.33** | -.29** |
| PCD conservation | -.38** | -.22* | -33** |
| PCD openness | -.13* | -.10 | -.05 |
| PCD self-enhancement | .04 | .11 | .09 |
| *R*^adj^*^2^* | .43** | .33** | .37** |
| **Polish migrants** |  |  |  |
| PCD self-transcendence | .06 | -.02 | -.02 |
| PCD conservation | -.41** | -.27** | -.43** |
| PCD openness | -.11 | .18* | .05 |
| PCD self-enhancement | -.02 | -.23* | -.14 |
| *R*^adj^*^2^* | .16** | .15** | .21** |

*Note. PCD is an abbreviation for perceived cultural distance which are difference-scores* *(host-society values minus migrant values). β is the standardized regression coefficient.* **p* < .05. ** *p* < .01.

**Table 14.** Study 2: Pearson correlations between PCD in the four types of values.

|  | **Correlations** | | | |
| --- | --- | --- | --- | --- |
|  | 1 | 2 | 3 | 4 |
| 1. PCD self-transcendence | - |  |  |  |
| 2. PCD conservation | .77** | - |  |  |
| 3. PCD openness | .46** | -.26** | - |  |
| 4. PCD self-enhancement | -.12** | -.06** | .31** | - |

*Note.* PCD is an abbreviation for perceived cultural distance which are difference-scores (host-society values minus migrant values). **p* < .05. ***p* < .01. ^†^*p’s* = .09.

**Response Surface Plots**

Plots of the response surface analysis (part of the polynomial regression) are presented below. Due to the amount of analyses, we only reported the plots for migrant attitudes. The plots for policy support and tolerance in Study 2 for the social values were consisted with the plots for migrant attitudes. The plots for personal values varied. The scales of the values are centred. Please note, that the plots do not take error estimates or *p*-values into account, hence, effects may be non-significant. Consult the statistics reported in the manuscript and the tables above for the interpretation of the plots. Colours in the graph is only intended to help with the three-dimensional interpretation and does not represent any meaning.

|  |  |
| --- | --- |
|  |  |

**Figure 1.** Response surfaces of perceived cultural distance in the Moroccan migrant condition of Study 1.

|  |  |
| --- | --- |
|  |  |

**Figure 2.** Response surfaces of perceived cultural distance in the Syrian migrant condition of Study 1.

|  |  |
| --- | --- |
|  |  |

**Figure 3.** Response surfaces of perceived cultural distance in values in the Polish migrant condition of Study 1.

|  |  |
| --- | --- |
|  |  |

**Figure 4.** Response surfaces of perceived cultural distance in the Moroccan migrant condition of Study 2.

|  |  |
| --- | --- |
|  |  |

**Figure 5.** Response surfaces of perceived cultural distance in the Syrian migrant condition of Study 2.

|  |  |
| --- | --- |
|  |  |

**Figure 6.** Response surfaces of perceived cultural distance in the Polish migrant condition of Study 2.
